# Loss of exosomal miR-3188 in cancer-associated fibroblasts contributes to HNC progression

**DOI:** 10.1186/s13046-019-1144-9

**Published:** 2019-04-08

**Authors:** Xiaoning Wang, Xing Qin, Ming Yan, Jianbo Shi, Qin Xu, Zhihui Li, Wenjun Yang, Jianjun Zhang, Wantao Chen

**Affiliations:** 1 Department of Oral and Maxillofacial-Head and Neck Oncology, Ninth People’s Hospital, Shanghai Jiao Tong University School of Medicine, Shanghai, 200011 People’s Republic of China; 2 Shanghai Key Laboratory of Stomatology & Shanghai Research Institute of Stomatology, National Clinical Research Center of Stomatology, Shanghai, 200011 People’s Republic of China

**Keywords:** Cancer associated fibroblasts, Exosomes, miR-3188, Head and neck cancer

## Abstract

**Background:**

Head and neck cancer (HNC) is one of the most common deadly diseases worldwide. An increasing number of studies have recently focused on the malignant functions of cancer-associated fibroblasts (CAFs) in numerous cancers. However, the underlying mechanisms by which CAF-derived exosomes promote tumor progression need to be further elucidated. This study aims to determine whether the loss of specific miRNAs in CAF-derived exosomes may be involved in the malignant transformation of HNC.

**Methods:**

MiRNA array and real-time PCR assays were used to analyze the differential expression of miRNAs in exosomes from normal fibroblasts (NFs) and CAFs. Cell proliferation, EdU incorporation, colony formation, apoptosis, cell cycle distribution and xenograft assays were performed to examine the effects of miR-3188 on HNC in vitro and in vivo. Real-time PCR, western blotting and luciferase reporter assays were used to identify the target genes of miR-3188. Furthermore, tumor-bearing mouse models were used to prove the potential therapeutic value of miR-3188-loaded exosomes in HNC.

**Results:**

Our results showed that miR-3188 expression is reduced in exosomes and their parental CAFs from HNC tissues. In addition, miR-3188 can be transferred from fibroblasts to HNC cells by exosomes. Further exploration demonstrated that exosomal miR-3188 can influence the proliferation and apoptosis of HNC cells by directly targeting B-cell lymphoma 2 (BCL2) in vitro and in vivo. More importantly, we also found that miR-3188-loaded exosomes significantly inhibited tumor growth in vivo*.*

**Conclusions:**

Our findings revealed that CAF-derived exosomes contain lower miR-3188 levels than NFs, and the loss of miR-3188 in exosomes contributes to the malignant phenotypes of HNC cells through the derepression of BCL2. Furthermore, these data suggest the potential therapeutic value of exosomal miR-3188 for inhibiting HNC growth.

**Electronic supplementary material:**

The online version of this article (10.1186/s13046-019-1144-9) contains supplementary material, which is available to authorized users.

## Introduction

With more than 600,000 new cases each year, head and neck cancer (HNC) is one of the most common malignancies worldwide [1]. Despite advancements in multimodality treatment, including surgery, radiation, and chemotherapy, the mortality rates of HNC are still undesirable [2]. To improve the dismal outcomes, a better understanding of the underlying biological mechanisms of HNC progression is urgently needed. Previous research on tumor biology has focused predominately on cancer cell characteristics. However, the properties of tumor cells could also be affected by complex cell-cell interactions between cancer cells and the surrounding microenvironment [3, 4].

The tumor microenvironment (TME) is composed primarily of fibroblasts, extracellular matrix, endothelial cells and immune cells. As a predominant component of the TME, cancer-associated fibroblasts (CAFs) are widely reported to promote cancer development [5–8]. Accumulating studies have revealed that CAFs are not just a collection of surrounding cells without malignant functions; rather, CAFs contribute to tumor proliferation, invasion, and metastasis via their secretion of various growth factors, cytokines and chemokines and their degradation of the extracellular matrix [7, 9, 10]. Additionally, most recent studies have reported that CAFs can modulate the characteristics of tumor cells via exosomes [11–13]. Exosomes (30–150 nm) are small membrane vesicles of endocytic origin that are secreted by most cell types and contain cell-specific collections of proteins, lipids, and genetic material [14]. As an important signaling mediator, exosomes are involved in various cellular functions and disease states [15]. However, the role of CAF-derived exosomes in HNC progression is still unclear.

As a major class of small non-coding RNAs, miRNAs can mediate gene silencing at the post-transcriptional level by binding to the 3′-untranslated region (UTR) or open reading frame (ORF) of genes. miRNAs are widely recognized to exert important effects on the malignant transformation of tumor cells, and the dysregulation of miRNAs is regarded as a hallmark of HNC [16, 17]. As a crucial component of exosomes, miRNAs can be transported into recipient cells, thus influencing the crosstalk between tumor cells and non-tumor cells in the TME [18–21]. An increasing number of studies have demonstrated that miRNAs can be delivered from CAFs to tumor cells via exosomes; this process promotes tumor invasion, metastasis and chemoresistance. It has been reported that miR-9, which is upregulated in breast cancer cells, could be secreted by fibroblasts via exosomes; this miR-9 secretion stimulates tumor cell migration by reducing E-cadherin expression [13]. Another study indicated that CAF-derived exosomal miR-146a promoted the proliferation and gemcitabine resistance of tumor cells in pancreatic cancer [20]. Additionally, Chi et al. discovered that the exosomal transfer of stroma-derived miR-21 conferred paclitaxel resistance in ovarian cancer cells by targeting APAF1 [12]. The above data reveal that CAFs could functionally modulate the biological behaviors of tumor cells by delivering oncogenic miRNAs via exosomes. However, the loss of specific miRNAs in the exosomes of CAFs can also contribute to tumor progression. Zhang et al. found that the loss of exosomal miR-320a in CAFs contributed to hepatocellular carcinoma (HCC) proliferation and metastasis through the derepression of PBX3 [22]. Nevertheless, how the loss of specific exosomal miRNAs in CAFs affects the properties of HNC has not been well studied.

In the present study, we identified differentially expressed miRNAs in exosomes derived from CAFs isolated from HNC tissues and found that miR-3188 was the most downregulated miRNA in the CAF-derived exosomes. Next, we verified that reduced miR-3188 levels in CAF-derived exosomes increased proliferation and inhibited apoptosis in HNC cells by derepressing BCL2 expression in the recipient cells both in vitro and in vivo. Furthermore, we also observed that miR-3188-loaded exosomes could retard tumor growth in a mouse model of HNC. Altogether, these results indicate a new promising therapeutic strategy for HNC.

## Materials and methods

### Ethics statement

HNC tissue samples were obtained from the Ninth People’s Hospital of the Shanghai Jiao Tong University of Medicine. All participants provided written informed consent. The protocols were approved by the Clinical Research Ethics Committee of the Ninth People’s Hospital, and the research was performed according to the provisions of the Helsinki Declaration. The animal experiments were approved by the Shanghai Jiao Tong University Institute Animal Care and Use Committee.

### Patients and specimens

All clinical samples, including tissues and plasma, were obtained from the Department of Oral and Maxillary-Head and Neck Oncology, Ninth People’s Hospital, Shanghai Jiao Tong University of Medicine (Shanghai, China). The samples used in this study included total RNA from 115 HNC tissues and 102 normal oral epithelial tissues; 64 pairs of HNC tissues and adjacent normal tissues; 86 plasma samples from HNC patients and 36 plasma samples from healthy individuals; and 35 pairs of post-operation and pre-operation plasma samples from HNC patients.

For details, 64 pairs of tumor and adjacent normal tissues were obtained from patients who were diagnosed with primary HNC and underwent initial surgery between August 2016 and October 2017. The samples were quickly frozen in liquid nitrogen and stored until total RNA extraction was performed. A separate cohort of 115 patients was assembled from a large pool of patients in the database based on histological diagnosis of HNC between April 2003 and April 2007. In parallel, 102 specimens of normal oral epithelial tissues obtained from patients without HNC were used as control. We also retrospectively reviewed the medical records of patients. In addition, plasma samples were collected 1 day before surgery, and 1 week after surgery.

### Cell cultures

The human HNC cell lines HN4 and HN30 were kindly provided by the University of Maryland Dental School, USA. CAL 27, 293 T and MC-3 T3-E1 cells were purchased from the American Type Culture Collection (ATCC, USA). Human primary fibroblasts were isolated from tumors (CAFs) and adjacent normal tissues (NFs) from HNC patients by primary culture and were identified by the presence of CAF-specific markers (α-SMA, FSP1, FAP) [23]. All these cells except MC-3 T3-E1 cells were cultured in Dulbecco’s modified Eagle’s medium (DMEM; Gibco-BRL, USA) supplemented with 10% fetal bovine serum (FBS; Gibco-BRL), penicillin (100 units/mL), and streptomycin (100 μg/mL) at 37 °C in a humidified 5% CO_2_ atmosphere, while MC-3 T3-E1 cells were maintained in MEM-α medium containing 10% FBS. All cell lines have been authenticated by short tandem repeat technology (STR) and tested for mycoplasma contamination.

### Immunofluorescence

For immunofluorescence studies, cells were fixed in 4% paraformaldehyde and permeabilized with 0.2% Triton X-100, followed by blocking with 5% goat serum. The samples were incubated with specific primary antibodies overnight at 4 °C, then incubated with the fluorescent secondary antibody (Life Technologies, USA). Cellular nuclei were counter stained with DAPI (Roche, USA). All of the labeled cells were examined using a Leica confocal fluorescence imaging microscope with LAS AF software, version 2.0 (Leica Microsystems, Germany). Antibodies used in this study are summarized in Additional file 1: Table S1.

### Immunoblotting analysis

The cells were harvested at the indicated times and rinsed with PBS, and exosomes were collected as described below. Then, total protein was extracted using SDS lysis buffer (Beyotime, China). The protein samples were subjected to 4–20% polyacrylamide gels (Genshare biological, China) to electrophoresed and transferred to 0.22-μm polyvinylidene fluoride (PVDF) membranes (Merck Millipore, USA). After blocked with 5% skimmed milk for 1 h at room temperature, the membranes were incubated overnight at 4 °C with specific primary antibodies. GAPDH and β-tubulin were used as loading controls. Afterwards, the membranes were probed with relevant secondary antibodies (Cell signaling technology, USA; 1:30,000) labeled with IR Dyes. Specific antibody-bound protein bands were visualized using an Odyssey Infrared Imaging System (LI-COR Bioscience, USA). See Additional file 1: Table S1 for antibodies used.

### MiRNA array analysis

MiRNA expression profiling was performed with 3 pairs of NFs and CAFs using the Affymetrix miRNA array platform (oebiotech, Shanghai, China). The extracted miRNA samples were labeled with the FlashTag Biotin HSR RNA Labeling Kit (Affymetrix P/N 901910, Thermo Scientific, USA). After hybridization, the Affymetrix miRNA 3.0 array (Affymetrix) scanning was performed using the Affymetrix Scanner 3000 (Affymetrix, Thermo Scientific, USA) and the result were analyzed using Affymetrix GeneChip Command Console software (version 4.0, Affymetrix). The intensity of the green signal was calculated after background subtraction, and replicated spots on the same slide were averaged to obtain the median intensity.

### Exosome isolation and purification

The cells were cultured without FBS or penicillin-streptomycin for 48-72 h and exosomes were collected from the conditioned media (CM). Briefly, the CM was spun at 300×g for 5 min to remove dead and floating cells and 3000×g for 15 min to remove cell debris. Then the resulting supernatant was passed through a 0.22-μm PVDF membrane filters and was spun at 120,000×g for 70 min to pellet the exosomes using a Beckman Coulter Type 55.2 Ti rotor. Finally, the pellet was washed once with PBS and purified by ultra-centrifuged at 10,000×g using Amicon Ultra-15 Centrifugal Filter Devices (100 K; Merck Millipore, USA). The final pellet containing exosomes was re-suspended in PBS and used for further investigations.

### Transmission electron microscopy

For electron microscopy analysis, the isolated exosomes were re-suspended with low salt PBS and dropped onto 100-mesh copper grid. The samples were negatively stained by 2% phosphotungstic acid for 3 min, allowed to dry for 15–20 min and examined under the transmission electron microscope (FEI Tecnai G2 Spirit, Thermo Fisher Scientific) at 80 kV.

### Nanoparticle tracking analysis

Nanoparticle tracking analysis (NTA) was performed with NanoSight NS300 (Malvern) equipped with a fast video capture and NTA analytical software. Nanoparticles were illuminated by the laser, and their movements under Brownian motion were captured for 60 s.

### RNA extraction and real-time PCR analysis

Total RNA from tumor cells, fibroblasts and tissues was extracted with TRIzol Reagent (Takara, Japan), while total RNA from exosomes was extracted using the mirVana™ miRNA Isolation Kit (Life Technologies, USA) according to the manufacturer’s instructions. Then, cDNA was synthesized using the PrimeScript RT reagent Kit (Takara, Japan), and miRNA was synthesized using the miRcute Plus miRNA First-Strand cDNA Synthesis Kit (Tiangen, China). The real-time PCR reactions for cDNA were performed using an ABI StepOne real-time PCR system (Applied Biosystems, USA) and the SYBR Premix Ex Taq Reagent Kit (Takara, Japan). MiRcute Plus miRNA qPCR Detection Kit (Tiangen, China) was used for the miRNA real-time PCR reactions. The reactions were performed according to the manufactures’ instruction. The relative gene expression was quantified using 2^-ΔΔCt^ method with logarithm transformation. β-actin or U6 snRNA was used as a control. The PCR primers are described in Additional file 2: Table S2.

### RNA extraction from plasma

MiRNA extraction from the samples of plasma and cell culture media was performed using the miRNeasy Mini Kit (Qiagen, Valencia, CA, USA). Briefly, plasma or culture media samples was thawed on ice and high centrifuged at 100,000 rpm for 15 min to remove cellular debris completely. Then 100 μL supernatant was lysed with 5 volumes of QIAzol solution. For normalization of sample-to-sample variation during the RNA isolation procedures, 25 fmol synthetic *Caenorhabditis elegans* miRNA-39 (syn-cel-miR-39) was added to each denatured sample. Small RNAs were enriched and purified according to the manufacturer’s protocol, followed by being eluted in 40 μL RNase-free water.

### Co-culture experiments

The co-culture experiments were divided into the transwell co-culture experiments and non-transwell ones. For the transwell co-culture experiments, tumor cells were seeded in the bottom well and CAFs were seeded on the upper insert (tumor: CAFs 1:3 ratio), and for the mixed co-culture experiments, tumor cells were mixed with an equal number of CAFs or NFs in 24-well plate. Co-cultures were maintained for 48 h for further experiments.

### Plasmid construction

To obtain the luciferase reporters, PCR-derived fragments from BCL2 3’UTR containing the miR-3188 binding site were inserted into the pmirGLO control vector (Promega, USA). Site-directed mutagenesis of the miR-3188 binding site in the BCL2 3’UTR was performed using GeneTailor Site-Directed Mutagenesis System. SV40, which encodes *Renilla* luciferase, was inserted in the vectors to normalize transfection efficiency.

The full-length sequences of BCL2 gene were amplified using PCR methods by a set of primers (forward primer: CCGGA ATTCG CCACC ATGGC GCACG CTGGG AGAA; reverse primer: CCGCT CGAGT CACTT GTGGC CCAGA TAGGC ACC). The amplified product of the BCL2 gene was purified, digested and ligated into the respective BanHI and EcoRI sites in the PGMLV-6395 vector (Genomeditech, China).

### Lentivirus production

For lentivirus package, miR-3188-expression vector was co-transfected with the GM easy™ Lentiviral Plasmid Mixture (Genomeditech, China) into 293 T cells using Lipofectamine 2000 reagent (Invitrogen, USA). In detail, the virus-containing supernatants were collected at 48 and 72 h after transfection and filtered using a 0.45 μm cellulose acetate filter (Merck Millipore, USA). Then the supernatants were diluted 2 times with serum-free DMEM containing polybrene (YEASEN, China) whose final concentration was 10 μg/mL. The mixed solutions were added to the tumor cells for another 8-h incubation before exchanged with fresh DMEM culture medium. After another 48-h incubation, the stably transfected cells were selected with 10 μg/mL puromycin (Sigma, USA).

### Cell transfection

Specific siRNA for BCL2, miR-3188 mimics and inhibitor were synthesized by Genomeditech Co. Ltd. (Shanghai, China). The sequences were shown in Additional file 3: Table S3. For transient transfection, HNC cells were seeded in a 6-well plate at 30–50% confluence. SiRNA and miRNAs were transfected at a working concentration of 50 nM using Lipofectamine 2000 according to the manufacture’s protocols. Cells were collected after 24-48 h for further experiments.

### Cell proliferation assay

MTT assay was performed to examine the cell viability. Tumor cells (1000/well) transfected with miR-3188 mimics, miR-3188 inhibitor, siRNA for BCL2, or BCL2-expressing plasmid in advance were seeded in 96-well plates. The cells were cultured for 1, 2, 3, 4, 5, 6 days. Subsequently, 10 μL of MTT (5 mg/mL in PBS; YEASEN, China) was added to each well and incubated for 4 h. The formazan crystals formed by viable cells were solubilized in 100 μL dimethyl sulfoxide (DMSO; MP Biomedicals, USA) and then the absorbance value (OD) was measured at 490 nm.

### Colony formation assay

Tumor cells (500/well) transfected with miR-3188 mimics and its inhibitor, siRNA for BCL2, or BCL2-expressing plasmid in advance were seeded in 6-well plates, and cultured in DMEM supplemented with 10% FBS for 7–10 days. Then, the colonies were washed twice with PBS and fixed with 4% paraformaldehyde. The crystal violet solution was used to stain the fixed colonies. The colonies composed of more than 50 cells were counted under a microscope. All experiments had 3 biological replicates.

### Wound healing assay

HNC cells (500,000/well) were pretreated as indicated and seeded in 6-well plates. A 10-μL pipette tip was used to create a wound field when the cells were grown to approximately 80% confluence. Then, the cells were washed with PBS and incubated with serum-free DMEM. Pictures of 5 non-overlapping fields were taken at 0 h and 16 h.

### Transwell migration and invasion assay

Transwell migration assay was performed with transwell chambers (pore 0.8 μm, Merck Millipore, USA). The cells (50,000/well) were suspended in 200 μL of serum-free medium and plated into the upper chambers. The lower chambers were filled with 600 μL medium plus 10% FBS as a chemoattractant. For transwell invasion assay, the transwell membrane was coated with 50 μL Matrigel (Corning, USA) in advance and allowed to dry for 2 h at 37 °C. After incubated for 24 h, the penetrated cells were fixed with 4% paraformaldehyde and stained with crystal violet. Cells on the upper surface of the filter were removed by wiping with a small cotton swab. Cells from five random non-overlapping fields were counted at × 200 original magnification.

### EdU incorporation assay

For EdU (5-ethynyl-2′-deoxyuridine) incorporation assay, proliferating HNC cells were examined using the Cell-Light EdU Apollo 488 In vitro Imaging Kit (Ribobio, China) according to the manufacture’s protocol. Briefly, twenty-four hours after transfection, the cells were incubated with 50 μM EdU for 2 h at 37 °C. Then, the cells were fixed with 4% paraformaldehyde for 30 min, followed by addition of 50 μL glycine (2 mg/mL, Yeasen, China). After 5 min, the cells were treated with 0.5% Triton X-100 (Sigma, USA) for 10 min at room temperature. Following washing with PBS for 5 min, 1 × Apollo reaction reagent was added and incubated at room temperature in the dark for 30 min, and then the cells were stained with 100 μL DAPI (1 μg/mL, Sigma, USA) for an additional 20 min in the dark. The number of EdU-positive cells was counted under a fluorescent microscope in 5 random fields. All experiments had 3 biological replicates.

### Luciferase assay

The luciferase-expressing HNC cells were co-cultured with fibroblasts and the cell samples were harvested for the detection of luciferase activity 48 h after co-culture. Cells were lysed in 120 μL lysis buffer (Yeasen, China). To measure firefly luciferase activity, 100 μL lysates was mixed with 100 μL of luciferase assay reagent (Yeasen, China), and then the relative luciferase units (RLU) was read with luminometer (Modulus, USA) immediately. The luciferase assay was performed three times in triplicate.

### Apoptosis analysis

To analyze apoptosis rates, the cells were digested with trypsin (BD Biosciences, USA) and resuspended as single-cell suspensions at 48 h after transfection. Approximate 1 × 10^6^ cells were incubated in 5 μL of FITC-Annexin V and 5 μL of propidium iodide (PI) for 15 min at 25 °C in the dark, after which the cells were diluted in another 400 μL of 1 × Binding Buffer and were analyzed immediately by flow cytometry (BD FASC Calibur, USA).

### Cell-cycle distribution analysis

HNC cells were transfected as indicated. About 24 h later, the cells were starved for 12 h in serum-free DMEM to synchronize, then harvested during the logarithmic growth phase after another 12 h incubation. Single-cell suspensions were fixed in 75% precooling ethanol overnight at 4 °C, washed twice with PBS, and incubated with 500 μL PI/RNase staining buffer (BD Biosciences, USA) for 30 min in dark. Cells were then evaluated using flow cytometry (BD FASC Calibur, USA), and the cell cycle distribution was analyzed with Cell Quest Software (BD Biosciences, USA).

### Luciferase reporter assay

HN4 and HN30 cells were seeded onto 24-well plates 24 h before transfection. The cells were transfected with 0.4 μg of the BCL2 reporter construct containing the predicted miR-3188 binding sites, together with 30 nM miR-3188 mimics or anti-miR-3188 using Lipofectamine 2000. Twenty-four hours after transfection, the firefly and Renilla luciferase signals were determined using the Dual Luciferase Reporter Assay System (Promega, USA) according to the manufacturer’s instructions. To investigate the effect of fibroblast-CM on the luciferase activity of BCL2 reporter, HN4 cells were transfected with BCL2 reporter. Six hours after transfection, HN4 cells were incubated with fibroblast-CM for 24 h and then the luciferase activity was assessed.

### Tumorigenicity assay in vivo

To evaluate the effect of miR-3188 loss on tumorigenicity in vivo, an HNC xenograft model was used. Male BALB/C nude mice aged 30–35 days and weighing approximately 20 g (purchased from Shanghai Laboratory Animal Center) were used for the in vivo experiments. All mice were maintained in autoclaved filter-top micro-isolator cages with autoclaved water and sterile food. Animal welfare and experimental procedures were conducted in compliance with the Guide for Care and Use of Laboratory Animals (Ministry of Science and Technology of China, 2006) and the related ethical regulations of the hospital. Briefly, a total of 1 × 10^6^ miR-NC-expressing CAL 27 cells or miR-3188-expressing CAL 27 cells in 100 μL serum-free DMEM were subcutaneously injected into the left and right buttocks of the mice.

To further study the effects of the loss of exosome-transmitted miR-3188, the mice were divided into 3 groups and injected with 100 μL serum-free DMEM containing the following cells: 5 × 10^5^ CAL 27 cells mixed with 5 × 10^5^ NFs, 5 × 10^5^ CAL 27 cells mixed with 5 × 10^5^ anti-miR-3188-expressing NFs, or 5 × 10^5^ CAL 27 cells mixed with 5 × 10^5^ NFs pretreated with GW4869 (10 μM). Three days after the injections, the GW4869-pretreated group was injected intraperitoneally with GW4869 (2 mg/kg body weight) every 48 h during the experiment. Mice were sacrificed 24 h after the final injection [24].

For the exosome treatment experiment, we established another xenograft model. Briefly, a total of 1 × 10^6^ CAL 27 cells in 100 μL serum-free DMEM were subcutaneously injected into the left and right flanks of male BALB/C nude mice. After 1 week, the tumor sizes were determined using micrometer calipers, and nude mice with similar tumor volumes were divided randomly into three groups. Exosomes were isolated from MC-3 T3-E1 cells and measured by Nanosight™ analysis. To load the exosomes with miR-3188, we mixed exosomes with cholesterol-conjugated miR-3188 under the specific conditions described before [25]. Briefly, 1 × 10^9^ exosomes were resuspended in PBS and then incubated with cholesterol-conjugated miRNA (cc-miRNA) at a ratio of 1 exosome: 15 cc-miRNA molecules in 100 μL at 37 °C for 1 h. After incubation, the exosomes were kept on ice and injected immediately into the mice. Intra-tumor injections of 100 μL of the exosome, exo-miR-NC or exo-miR-3188 suspension were performed for each tumor every 2 days for 19 days.

No obvious health problems were observed during treatment. Tumor volumes were measured every 3 days using the following formula: tumor volume = length × width^2^/2. Tumor growth curves were plotted using the seeding day as the horizontal axis and the tumor volume as the vertical axis.

### Immunohistochemical analysis

Paraffin-embedded sections prepared from the in vivo experiments were used for immunohistochemistry assays to detect the protein expression levels of Ki-67 and BCL2. Briefly, the sections were deparaffinized, rehydrated, submerged in citrate antigen retrieval solution (Solarbio, China) for heat-induced antigenic retrieval, immersed in 0.3% hydrogen peroxide to block endogenous peroxidase activity, blocked with 5% goat serum, incubated with primary antibody at 4 °C overnight and developed using a DAKO ChemMate Envision Kit/HRP (Dako-Cytomation, USA). Then, the sections were counterstained with hematoxylin, dehydrated, cleared and mounted with neutral gums. Protein expression was determined by randomly selecting three tumor cell areas for each specimen under the same conditions using Image-pro plus 6.0 software. The mean optical density (MOD) was calculated with the following formula: MOD = IOD SUM / area SUM (IOD: the integrated optical density; IOD SUM: the cumulative IOD of the targeted areas in one photo; area SUM: the sum of the targeted areas). In addition, PBS was substituted for the primary antibody for the blank control. See Additional file 1: Table S1 for the antibodies used.

### TUNEL assay

Paraffin-embedded sections were deparaffinized and rehydrated as described above. Then, apoptosis was assessed using a terminal deoxynucleotidyl transferase dUTP nick and labeling (TUNEL; Boster, China) assay according to the manufacturer’s instructions. Briefly, endogenous peroxidase activity was quenched by incubation with 0.3% hydrogen peroxide at 37 °C for 10 min. Then, the sections were treated with proteinase K diluted 1:200 in TBS for 5 min at 37 °C in a humidified chamber. A labeling mixture containing digoxin-dUTP in a terminal deoxynucleotidyl transferase (TdT) enzyme buffer was added to the sections and incubated at 37 °C for 2 h. After washing thrice with TBS for 2 min, the sections were covered with anti-digoxin-biotin conjugate diluted 1:100 in blocking reagent and incubated at 37 °C for 30 min. The tissues were then incubated for 1 h at 37 °C with streptavidin-biotin complex (SABC) diluted 1:100 in TBS. Labeling was visualized with 3′3’-diaminobenzidine. The sections were then counterstained with hematoxylin. For the negative control, representative sections were processed in the same way, but incubation with the TdT enzyme buffer was omitted.

### Statistical analysis

Statistical analyses were performed using Statistical Package for Social Science software Version 16.0 (SPSS 16.0). All quantitative experiments were repeated with at least three independent biological repeats and are presented as the means ± SD (standard deviation). Quantitative data were analyzed by either one-way analysis of variance (ANOVA) (multiple groups or parametric generalized linear model with random effects for tumor growth and MTT assay) or t-test (two groups). The Mann-Whitney U-test was used to analyze the associations between BCL2 mRNA or miR-3188 levels and clinical parameters. The correlation between miR-3188 and BCL2 was determined by Pearson analysis. The log-rank test was used to assess the survival differences and Kaplan-Meier survival analyses were used to estimate the prognostic and diagnostic value. A *p*-value < 0.05 (two-sided) was considered statistically significant.

## Results

### MiR-3188 is significantly reduced in the exosomes of CAFs derived from HNC patients

**Fig. 1:**
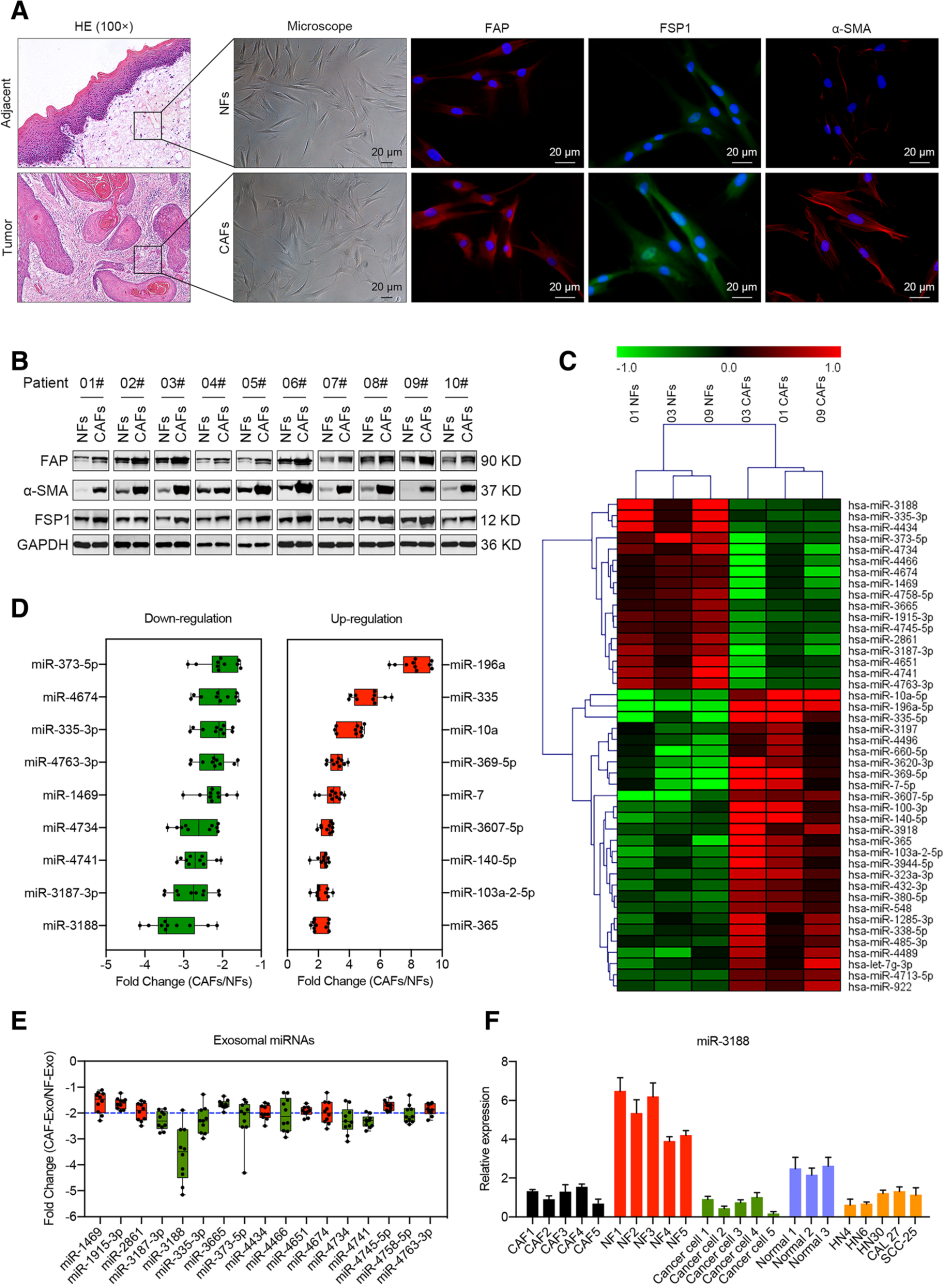
Loss of miR-3188 in exosomes of CAFs derived from HNC patients. **a** The morphological characteristics of NFs and CAFs derived from HNC and adjacent normal tissues (Left), immunofluorescence analysis of the expression of CAF markers (FAP, FSP1 and α-SMA) in isolated NFs and CAFs (Scale bar = 20 μm). **b** Immunoblotting analysis of the expression of FAP, α-SMA and FSP1 in 10 paired of primary cultured NFs and CAFs. **c** Heatmap diagram of differential miRNA expression profiles of 3 paired of NFs and CAFs. The miRNA expression levels are hierarchically clustered on the y-axis, and CAFs or the paired NFs are hierarchically clustered on the x-axis. Red indicates up-regulation; green indicates downregulation. **d** Validation of the differential expression levels of the 18 randomly selected microRNAs in 10 paired of NFs and CAFs using real-time PCR. **e** Analysis of expression levels of 17 most downregulated microRNAs in exosomes derived from 10 paired of NFs and CAFs using real-time PCR. **f** Real-time PCR analysis of miR-3188 expression in cultured CAFs, NFs, cancer cells, normal cells and HNC cell lines

We first isolated CAFs and normal fibroblasts (NFs) from HNC tissues and adjacent normal tissues. As previously reported, the CAFs showed an elongated, mesenchymal morphology [9]. Using immunofluorescence and western blotting assays, we also identified high expression levels of the CAF-specific markers FAP, FSP1 and α-SMA in primary-cultured CAFs (Fig. 1a, b). In accordance with previous reports [5], further experiments demonstrated the promoting effects of CAFs on tumor proliferation, invasion, migration and chemoresistance (Additional file 4: Figure S1).

Exosomes, which are mediators of intercellular communication, could modify recipient cell functions by delivering specific signaling factors [26]. Given that CAFs facilitated HNC cell proliferation, migration and invasion, we speculated that exosomes play a crucial role in these processes. We then isolated exosomes from CAF-conditioned medium (CM) by ultracentrifugation and investigated the effects of these exosomes on HNC cells. The purified exosomes were identified by the positive expression of Alix, HSP90, CD63, CD9 and CD81 and the negative expression of GM130 [14, 27, 28] (Additional file 4: Figure S2a). Nanoparticle tracking analysis (NTA) was also used to determine the average size and concentration of the exosomes. The analysis established that exosomes from both CAFs and NFs have a similar in number and size-distribution within the typical range for exosomes (between 30 and 150 nm, Additional file 4: Figure S2b). In addition, transmission electron microscopy was used to detect spherical, membrane-encapsulated particles between 30 and 150 nm, sizes that are typical of exosome vesicles (Additional file 4: Figure S2c).

Consistent with the effects of CAFs on HNC cells, CAF-derived exosomes notably promoted the proliferation, migration and invasion of HN4 and HN30 cells when compared with NF-derived exosomes (Additional file 4: Figure S3a-d). Furthermore, to explore whether exosomes are required for these effects, we dislodged the exosomes by ultracentrifugation or reduced exosome production through the pharmacological inhibition of neutral sphingomyelinase-2 (nSMase) with GW4869. As shown in (Additional file 4: Figure S3e-g), exosome-depleted CAF-CM had little effect on the behaviors of recipient tumor cells. These results indicated the critical role of CAF-derived exosomes in facilitating tumor progression.

To investigate the underlying mechanism mediated by CAF-derived exosomes, we next focused on the dysregulation of miRNAs in CAFs. The miRNA expression levels of 3 pairs of NFs and CAFs were analyzed using a miRNA array. A heatmap showed a clear distinction between CAFs and NFs (Fig. 1c). Compared with the miRNAs in NFs, 17 miRNAs were downregulated, and 27 miRNAs were upregulated in CAFs (Additional file 5: Table S4). In addition, 18 randomly chosen miRNAs were assessed in another 10 paired NFs and CAFs using real-time PCR to further validate the expression data obtained by microarray hybridization (Fig. 1d). High consistency was found in these two assays. The data suggested that the miRNA array results can provide some valuable information. In an attempt to identify the most reduced miRNA in CAF-derived exosomes, we evaluated the 17 downregulated miRNAs in 10 paired CAF- and NF-derived exosomes by real-time PCR. Notably, miR-3188 was the most downregulated miRNA in the CAF-derived exosomes (Fig. 1e). Therefore, we selected miR-3188 for further investigation. We next performed a real-time PCR analysis of miRNA isolated from CAFs, NFs, primary HNC cells, primary normal oral epithelial cells and HNC cell lines. The results showed that NFs expressed significantly higher levels of endogenous miR-3188. Moreover, compared with that in normal epithelial cells, the expression of miR-3188 in cancer cells was reduced (Fig. 1f). Collectively, these data indicated that the loss of miR-3188 might be involved in tumor malignancy.

### Exosomal transfer of miR-3188 from fibroblasts to tumor cells

**Fig. 2:**
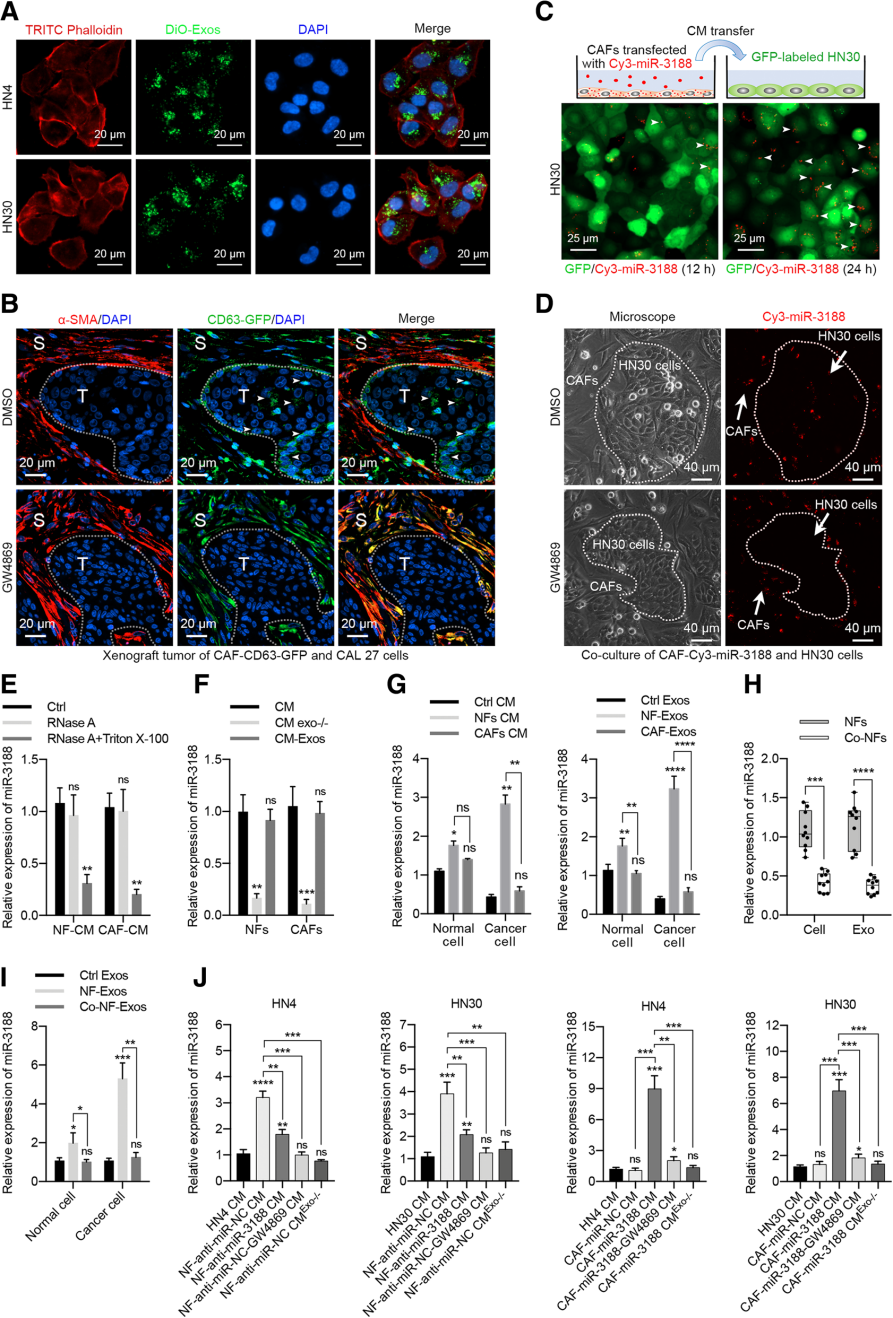
Exosomal transfer of miR-3188 from fibroblasts to HNC cells. **a** HN4 and HN30 cells were culture with DiO-labeled exosomes from CAFs for 24 h, and Laser Scanning Confocal Microscopy was used to analyze the internalization of CAF-derived exosomes into HNC cells (Scale bar = 20 μm). **b** A mixture of CAL27 cells and CAFs expressing CD63-GFP fusion protein were used to generate xenografts in nude mice, and the mice were treated with or without GW4869 (2 mg/kg). Immunofluorescence staining was used to detect the stroma cell-derived exosomes internalization by HNC cells in xenografts (S, stroma; T, tumor; Scale bar = 20 μm). **c** Conditioned medium derived from CAFs transfected with cy3-labelled miR-3188 was added to HN30 cells for 24 h, and the red signal was detected using Confocal Microscopy (Scale bar = 25 μm). **d** Light microscopy and fluorescent images of HN30 cells after co-culture with CAFs loaded with cy3-miR-3188 for 24 h (Scale bar = 40 μm). **e** NF-CM or CAF-CM were treated with RNase A (2 mg/ml) or/and 0.1% Triton X-100, miRNA in CM was isolated and miR-3188 was detected using real-time PCR. **f** miR-3188 expression levels in CM, CM dislodged with exosomes and exosomes derived from CM of NFs and CAFs. **g** Primary cultured normal epithelial cells and cancer cells were treated with CM (Left) or exosomes (Right) derived from control cells (Ctrl CM, Ctrl Exos), NFs or CAFs for 48 h, and miR-3188 was detected using real-time PCR. **h** The miR-3188 expressions in original NFs or NFs co-cultured with cancer cells (Co-NFs) were detected. **i** The normal cells and cancer cells were respectively treated with exosomes derived from control cells, original NFs or NFs co-cultured with cancer cells (Co-NF-Exos). Then the miR-3188 expression levels were detected. **j** After HNC cells were treated with CM of HNC cells, CM of fibroblasts with different miR-3188 expression or CM dislodged with exosomes, miR-3188 expressions were detected using real-time PCR. (ns, no significant difference, *p < 0.05, **p < 0.01, ***p < 0.001, ****p < 0.0001)

To determine whether CAF-derived exosomes were internalized by HNC cells, the purified exosomes were labeled with DiO and incubated with HN4 or HN30 cells for 24 h. As shown in Fig. 2a, DiO-labeled exosomes (green fluorescence) were observed in most recipient cells. CAFs stably expressing the CD63-GFP fusion protein were mixed with CAL 27 cells (1:1) to generate a xenograft to detect exosome internalization in vivo. Tumor-bearing mice were injected intraperitoneally with GW4869 (2 mg/kg body weight) or DMSO every 48 h for 3 weeks. An immunofluorescence assay revealed that GFP could be observed in both tumor cells and stromal cells in the DMSO-treated group but in only stromal cells in the GW4869-treated group (Fig. 2b). These data suggested that the CAF-derived exosomes could be internalized by tumor cells both in vitro and in vivo, and the administration of GW4869 inhibited the exosome transfer from stromal cells to tumor cells. Next, we investigated whether miR-3188 could be transferred from CAFs to HNC cells via exosomes. GFP-labeled HN30 cells were incubated with exosomes from CAFs transfected with cy3-tagged miR-3188 for 24 h. As shown in Fig. 2c, red fluorescent signals were observed over time. We also co-cultured HN30 cells with CAFs transfected with cy3-tagged miR-3188. Fluorescence microscopy revealed red fluorescence in the HN30 cells, but GW4869 treatment abolished cy3-oligo expression (Fig. 2d). To further investigate the existing pattern of extracellular miR-3188, NF-CM and CAF-CM were treated with RNase A and Triton X-100 [29]. The levels of miR-3188 in CM were unchanged upon RNase A treatment but significantly decreased when treated with RNase A and Triton X-100 simultaneously (Fig. 2e). These results suggest that extracellular miR-3188 was mainly wrapped by the membrane instead of being directly released. In addition, we also found that miR-3188 levels in exosomes of NFs or CAFs were almost equal to those in whole CM (Fig. 2f). Together, these data reveal that miR-3188 delivery from fibroblasts to tumor cells might occur mainly by exosomes.

Next, to determine the impact of fibroblast-derived exosomes on miR-3188 expression in tumor cells, we treated normal epithelial cells and cancer cells with NF-CM or CAF-CM. MiR-3188 expression levels were increased after NF-CM treatment, but no significant changes were observed upon CAF-CM treatment. Furthermore, similar miR-3188 expression changes were detected upon treatment with the respective derived exosomes. The average fold change of miR-3188 was higher in cancer cells than in normal cells, which could be due to the lower basal expression of miR-3188 in cancer cells (Fig. 2g). Besides, co-culture with cancer cells could reduce the expression of miR-3188 in NFs (Fig. 2h, i).

Moreover, CM of NFs with suppressed miR-3188 expression induced less miR-3188 augmentation in recipient cells. Conversely, CM of CAFs with increased miR-3188 expression significantly increased miR-3188 levels in HN4 and HN30 cells. As expected, the pharmacological or physical depletion of exosomes from fibroblast-CM abrogated these effects (Fig. 2j). Together, these data verified that the loss of miR-3188 in CAFs might downregulate miR-3188 in tumor cells. The preceding results raised the question of whether the loss of miR-3188 in CAF-derived exosomes could alter the biological features of HNC cells.

### MiR-3188 influences HNC cell proliferation and apoptosis

**Fig. 3:**
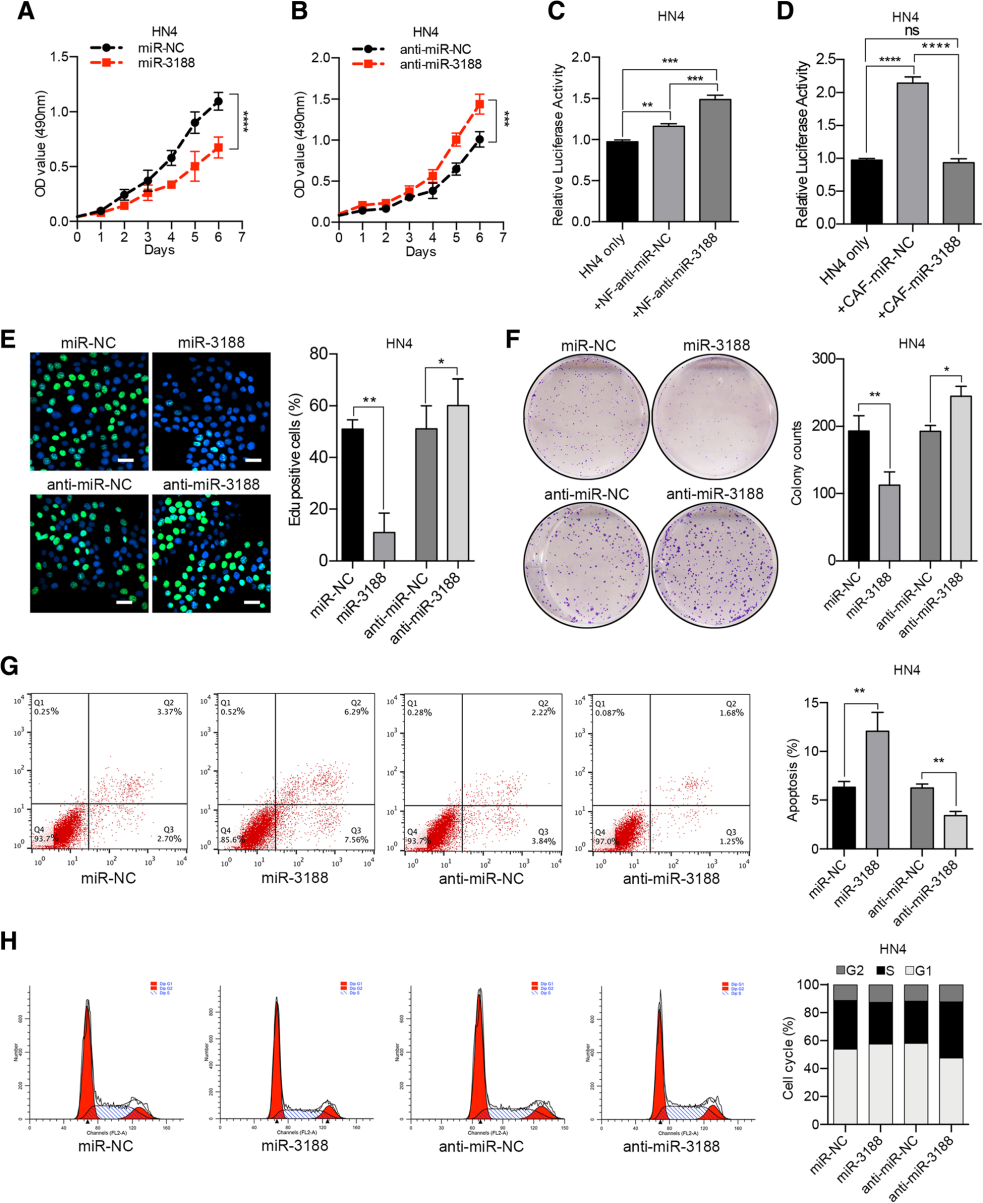
miR-3188 inhibits the growth of HN4 cells and facilitates the apoptosis of HNC cells in vitro. **a** Transfection of miR-3188 mimics inhibited the growth of HN4 cells by MTT assay. **b** miR-3188 downregulation increased the growth of HN4 cells by MTT assay. **c** NFs were transfected with miR-3188 inhibitor, and 24 h later NF-anti-miR-NC and NF-anti-miR-3188 were co-cultured with HN4 cells (1:1) for another 48 h, then the luciferase activity was assessed. **d** CAFs were transfected with miR-3188 mimics, and 24 h later CAF-miR-NC and CAF-miR-3188 were co-cultured with HN4 cells (1:1) for another 48 h, then the luciferase activity was assessed. EdU incorporation assay (**e**), colony formation assay (**f**), apoptotic analysis (**g**) and cell cycle analysis (**h**) of HN4 cells were performed after transfection with miR-3188 mimics or inhibitor as indicated. Scale bar = 40 μm. (NC, negative control. ns, no significant difference, *p < 0.05, **p < 0.01, ***p < 0.001, ****p < 0.0001)

In an attempt to determine the impact of miR-3188 on tumor cell proliferation, HN4 and HN30 cells were transfected with miR-3188 mimics and inhibitors (Additional file 4: Figure S4). Then, MTT assays were utilized to evaluate the proliferation of HN4 and HN30 cells. The overexpression of miR-3188 significantly inhibited HN4 and HN30 cell proliferation (Fig. 3a, Additional file 4: Figure S5a). In parallel, the suppression of miR-3188 enhanced HNC cell proliferation (Fig. 3b, Additional file 4: Figure S5b). We also co-cultured luciferase-expressing tumor cells with NFs, and luciferase activity was assessed. As expected, the suppression of miR-3188 in NFs accelerated HNC cell proliferation. Conversely, the overexpression of miR-3188 in CAFs abrogated this effect (Fig. 3c and d, Additional file 4: Figure S5c and d). Subsequently, we performed EdU incorporation assays, colony formation assays, apoptosis assays and cell cycle analyses to further examine the effects of miR-3188. The results showed that the overexpression of miR-3188 markedly suppressed cancer cell proliferation, colony formation ability and G1 to S cell cycle transition. In addition, the apoptosis ratio was increased. Conversely, decreased miR-3188 expression promoted the G1 to S cell cycle transition and colony formation ability and decreased the apoptosis ratio in HN4 and HN30 cells (Fig. 3e-h, Additional file 4: Figure S5e-h).

In addition to proliferation and apoptosis, we explored the effects of miR-3188 on the mobility and invasive phenotypes of HNC cells. Using wound healing and transwell invasion assays, we observed that miR-3188 had little effect on the migration and invasion of HN4 and HN30 cells (Additional file 4: Figure S6).

### BCL2 is a direct target of miR-3188 in HNC cells

**Fig. 4:**
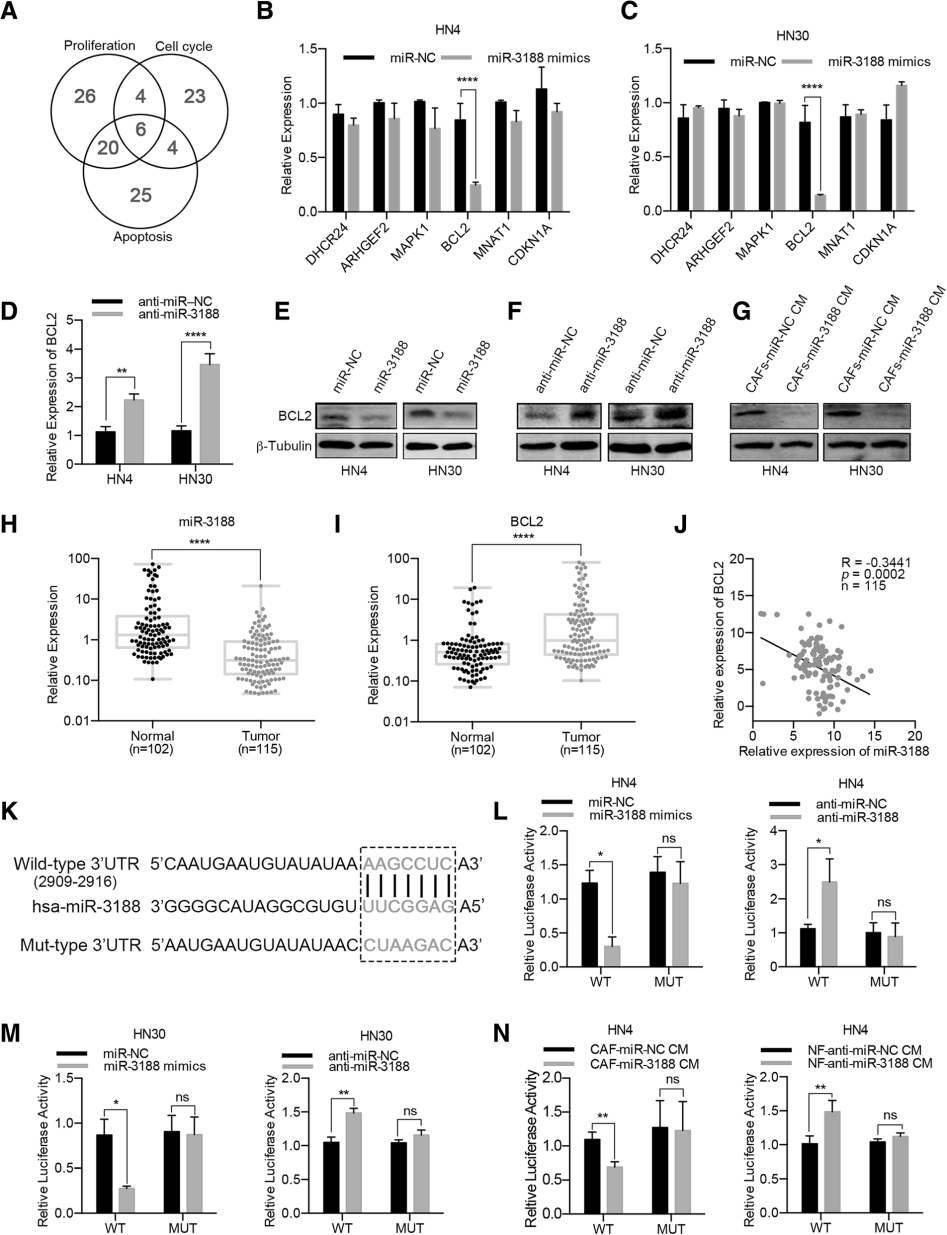
miR-3188 directly targets BCL2 in HNC cells. **a** The target genes of miR-3188 were predicted by bioinformatics analysis and then subjected to gene ontology analysis. Six candidate genes related to proliferation, cell cycle and apoptosis were screened for further verification. **b**, **c** After a 24 h transfection with miR-3188 mimics in HN4 and HN30 cells, the expression levels of potential targets for miR-3188 were analyzed using real-time PCR. **d** After a 24 h transfection with miR-3188 inhibitor in HN4 and HN30 cells, the BCL2 expression levels were analyzed using real-time PCR. **e**, **f** After a 48 h transfection with miR-3188 inhibitor in HN4 and HN30 cells, the BCL2 expression levels were analyzed using western blotting. **g** CAFs were transfected with miR-3188 mimics and CM was collected 48 h later. After treatment with CM of miR-3188 overexpressed CAFs for 48 h, BCL2 protein levels in HN4 and HN30 were analyzed using western blotting. **h**, **i** miR-3188 and BCL2 expression in 102 normal epithelial tissues and 115 HNC tissues were detected by real-time PCR. **j** Pearson analysis was performed in the above cohort and an inverse correlation was observed between the miR-3188 and BCL2 expression levels. **k** Predicted miR-3188 binding site in the 3’UTR region of BCL2. A mutated miR-3188 binding site was generated in the complementary site for the seed region of miR-3188. **l**, **m** An miR-3188 mimics or inhibitor and a luciferase vector encoding the wild-type or mutant BCL2 3′-UTR region were co-transfected into HN4 and HN30 cells, after another 24-h incubation the relative luciferase activity was measured. **n** HN4 cells was transfected with a luciferase vector encoding the wild-type or mutant BCL2 3′-UTR region, and treated with CM of CAFs overexpressed miR-3188 or CM of NFs silenced miR-3188, then the luciferase activity was measured. (NC, negative control. ns, no significant difference, *p < 0.05, **p < 0.01, ***p < 0.001, ****p < 0.0001)

The preceding data indicated that miR-3188 affected the proliferation, cell cycle transition and apoptosis of HNC cells. Therefore, we sought to identify the potential targets of miR-3188 that contribute to its antiproliferative and pro-apoptotic functions. We performed a bioinformatics analysis to search for potential targets of miR-3188 using TargetScan, miRWalk, miRanda and RNA22. Of all the genes, 919 were predicted by all four databases. The 919 predicted targets were then subjected to gene ontology analysis (http://david.abcc.ncifcrf.gov/). Among the putative candidates for miR-3188, there were 6 candidate genes whose functions were related to proliferation, cell cycle and apoptosis (Fig. 4a). After HN4 and HN30 cells were transfected with miR-3188 mimics, only the expression of BCL2 from the 6 candidate proteins was significantly repressed (Fig. 4b, c). In contrast, the downregulation of miR-3188 increased BCL2 expression levels in HNC cells (Fig. 4d). Western blot results revealed BCL2 protein downregulation in miR-3188-overexpressing cell lines and BCL2 protein upregulation in miR-3188-suppressed cells (Fig. 4e, f). In addition, treatment with CM from miR-3188-overexpressing CAFs obviously decreased the BCL2 protein levels in HN4 and HN30 cells (Fig. 4g).

To determine whether BCL2 is a direct target of miR-3188, we first quantified the miR-3188 and BCL2 mRNA levels in HNC cohorts using real-time PCR. Compared to that in normal oral epithelial tissues, miR-3188 was significantly downregulated in HNCs (Fig. 4h), while BCL2 mRNA was upregulated in the same samples (Fig. 4i). BCL2 expression was negatively correlated with miR-3188 expression in the same HNC cohorts (Fig. 4j). Then, we constructed a luciferase vector containing a wild-type or mutant 3’UTR region of BCL2 (Fig. 4k). HN4 and HN30 cells were co-transfected with the vectors and miR-3188 mimics or inhibitor, and luciferase activity was detected after 24 h. Compared with the mock control, the miR-3188 mimics remarkably reduced luciferase activity, while the miR-3188 inhibitor had the opposite effect. Mutations in the binding sites of BCL2, however, dramatically abrogated these effects (Fig. 4l, m). In addition, CM from fibroblasts transfected with miR-3188 mimics or inhibitor was also used for the reporter assay, and similar results were obtained when HN4 cells were treated with the CM (Fig. 4n). The above data demonstrated that exosomal miR-3188 suppressed BCL2 mRNA expression by directly targeting BCL2.

### MiR-3188 inhibits the proliferation and apoptosis of HNC cells through downregulating BCL2

**Fig. 5:**
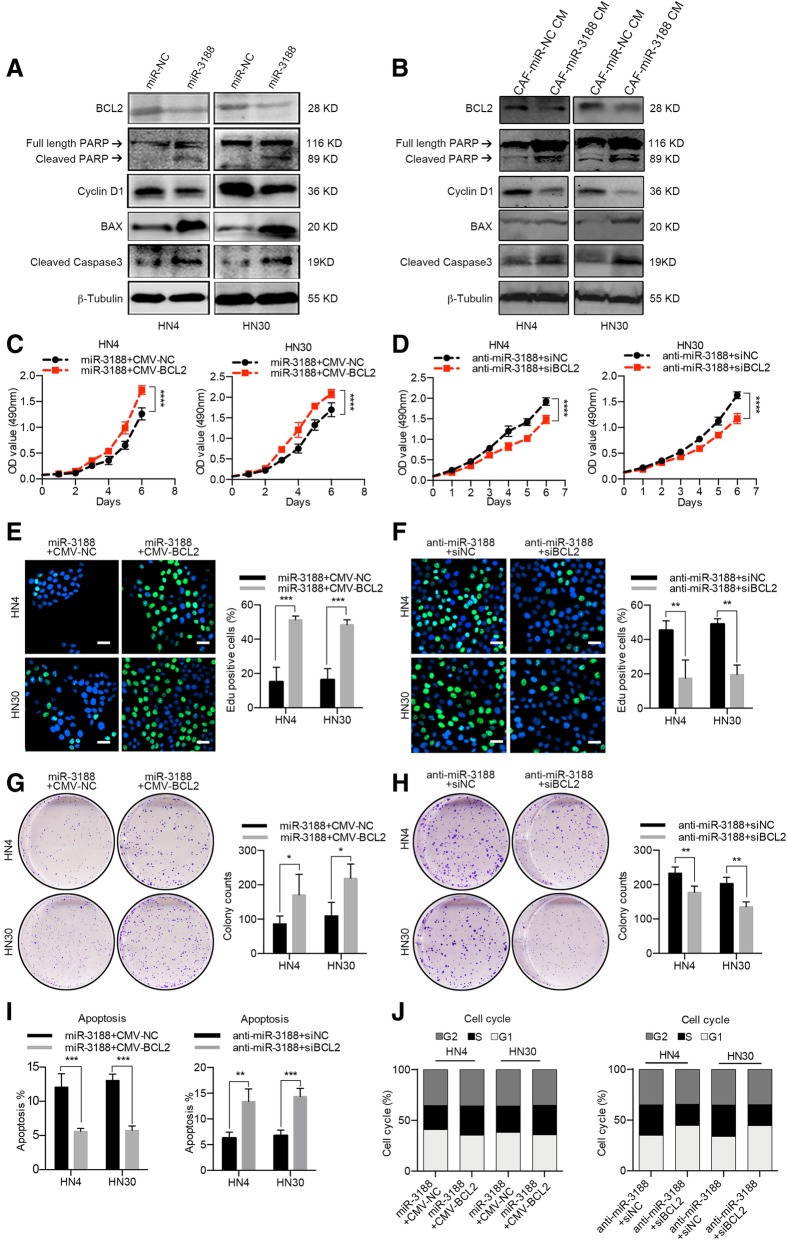
BCL2 overexpression reverse the effects of suppression of miR-3188. **a** Immunoblotting analysis of endogenous BCL2, cleaved PARP, cyclin D1, BAX and cleaved caspase3 protein expression levels in HN4 and HN30 cells treated with miR-3188 mimics. β-Tubulin served as a loading control. **b** CM was collected from CAFs overexpressed miR-3188 and used to treat HNC cells for 48 h, then BCL2, cleaved PARP, cyclin D1, BAX and cleaved caspase3 protein expression levels were assessed using western blotting. MTT assay (**c**, **d**), EdU assay (**e**, **f**), colony formation assay (**g**, **h**), apoptosis analysis (**i**) and cell cycle analysis (**j**) of HNC cells were performed after transfected with miR-3188 mimics, miR-3188 mimics plus ectopic BCL2, siBCL2 and siBCL2 plus miR-3188 inhibitor (Scale bar = 40 μm). (NC, negative control. *p < 0.05, **p < 0.01, ***p < 0.001, ****p < 0.0001)

Next, we investigated the effects of BCL2 on the biological properties of HNC cells. HN4 and HN30 were transfected with the specific siRNA or overexpression plasmid for BCL2 (Additional file 4: Figure S7a, b), then the proliferation, colony formation, apoptosis and cell-cycle assay were performed in HNC cells. Because BCL2 suppressed cell proliferation and colony formation and promoted cell apoptosis (Additional file 4: Figure S7c-h), we measured cyclin D1, cleaved PARP, BAX and cleaved caspase-3 protein levels in miR-3188-overexpressing cell lines. As shown in Fig. 5a, increased levels of cleaved PARP, caspase-3 and BAX were observed in miR-3188-overexpressing HNC cells, and the expression of cyclin D1 was reduced. Intriguingly, CM from miR-3188-expressing CAFs induced similar changes in protein expression (Fig. 5b). Moreover, we found that miR-3188-mediated BCL2 silencing resulted in reduced cyclin D1 levels, which indicated that BCL2 might be associated with cell cycle regulation. To exclude the effect of miR-3188, a specific siRNA for BCL2 was utilized to analyze cyclin D1 expression after BCL2 silencing. Interestingly, we observed that knocking down BCL2 decreased cyclin D1 levels, and the ectopic introduction of BCL2 enhanced cyclin D1 expression (Additional file 4: Figure S8).

Subsequently, we restored BCL2 expression by transiently transfecting with the full-length BCL2 plasmid in miR-3188-overexpressing HN4 and HN30 cells (Additional file 4: Figure S9). Transiently transfecting BCL2 into miR-3188-overexpressing HNC cells enhanced cell proliferation, colony formation and cell-cycle transition from G1 to S phase, as well as decreased the apoptosis rate. In parallel, BCL2 silence in miR-3188-downregulating HNC cells decreased cell proliferation, colony formation and G1 to S cell-cycle transition as well as promoted the apoptosis (Fig. 5c-j). Collectively, these results suggest that miR-3188 inhibits the proliferation and apoptosis of HNC cells through downregulating BCL2.

### Intercellular transfer of miR-3188 by exosomes inhibits tumor growth in vivo

**Fig. 6:**
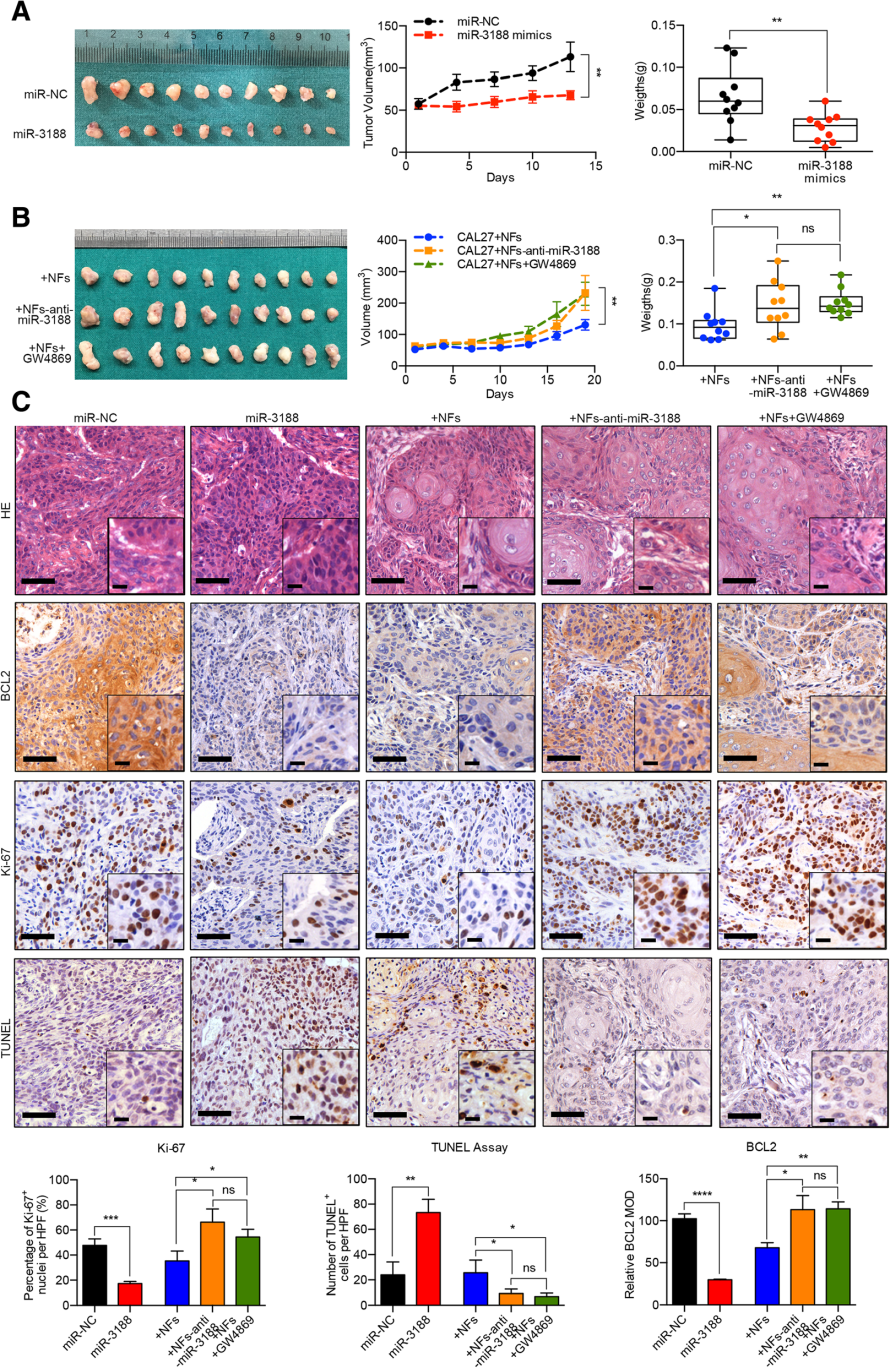
Intercellular transfer of miR-3188 by exosomes inhibits tumor growth in vivo*.* **a** MiR-3188-overexpressed CAL27 cells were utilized to generate xenografts in nude mice. The graphics of xenografts, tumor growth curve and tumor weights showed that miR-3188 inhibited the xenograft tumor growth in nude mice. **b** NFs silenced miR-3188 were mixed with CAL27 to generate xenografts. Besides, GW4869 was intraperitoneal injected to block the transportation of endogenous miR-3188 via exosomes. The data showed that suppression of miR-3188 in NFs or inhibition of exosome secretion by GW4869 retarded the xenograft tumor growth. **c** Immunohistochemical analysis of BCL2 expression, Ki-67 staining and TUNEL assay in the xenografts (Scale bar: main = 50 μm; insert = 20 μm). (NC, negative control. ns, no significant difference, *p < 0.05, **p < 0.01, ***p < 0.001, ****p < 0.0001)

After testing the effects of miR-3188 on tumor cell properties in vitro, we next analyzed whether miR-3188 or fibroblast-derived exosomes could affect tumorigenicity in vivo. CAL 27 cells stably expressing miR-3188 (Additional file 4: Figure S10a) were subcutaneously injected to generate xenografts in nude mice. We found that the mice injected with CAL 27-miR-3188 cells had a smaller tumor burden (Fig. 6a), which proved the inhibitory effect of miR-3188 on tumorigenicity. To further illustrate the effects of miR-3188 in NF-derived exosomes, NFs transfected with miR-3188 inhibitor or treated with GW4869 were mixed with CAL 27 cells, and an in vivo tumor formation experiment was conducted. During the experiment, the GW4869 treatment group received intraperitoneal injections of the inhibitor (2 mg/kg) every 48 h. The results showed that miR-3188 suppression in NFs (Additional file 4: Figure S10b) enhanced tumor growth. Importantly, a similar effect was obtained after exosome secretion was inhibited by GW4869 (Fig. 6b).

Subsequently, we evaluated BCL2 mRNA and protein expression in xenograft tissues using real-time PCR and IHC, respectively. Reduced BCL2 expression was detected in miR-3188-overexpressing CAL 27 xenografts, and suppressing miR-3188 expression in NFs or GW4869 treatment restored BCL2 levels in the tumors (Fig. 6c, Additional file 4: Figure S11). Ki-67 staining and TUNEL assays were performed to assess the proliferative activity of the xenografts. We observed decreased expression of the proliferative marker Ki-67 and increased TUNEL^+^ nuclei in the CAL 27-miR-3188 group. Compared with the CAL 27 + NF group, the CAL 27 + NF-anti-miR-3188 and CAL 27 + NF + GW4869 groups had improved Ki-67 expression and decreased TUNEL^+^ nuclei in cells (Fig. 6c). Together, these data suggested that the loss of miR-3188 in NF-derived exosomes was responsible for the enhanced tumor growth.

**Fig. 7:**
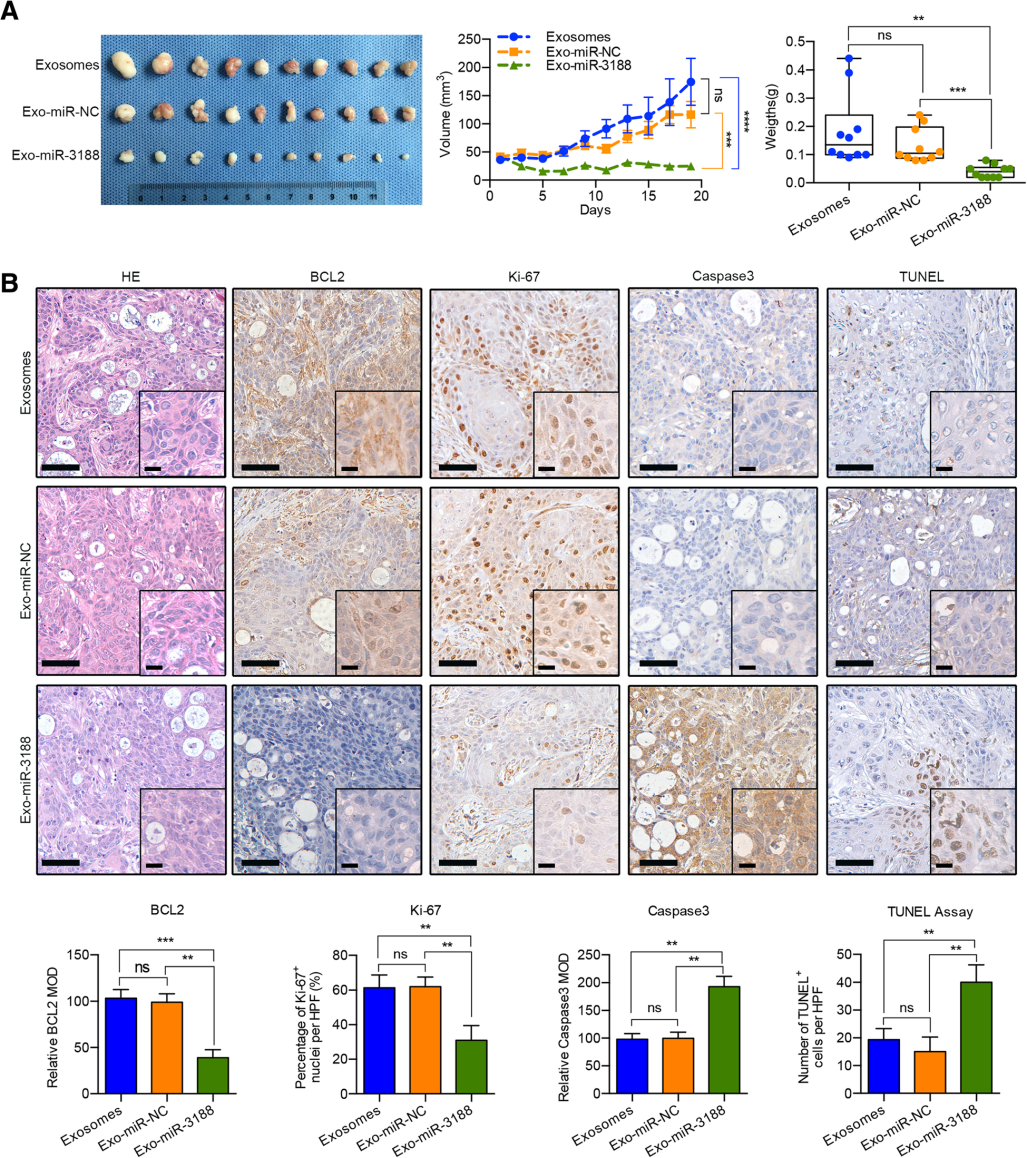
Intercellular transfer of miR-3188 by exosomes inhibits tumor growth in vivo*.* **a** Exosomes were loaded with miR-3188 mimics and used to treat the CAL27 xenografts-bearing mice. The graphics of xenografts, tumor growth curve and tumor weights showed that intra-tumor injection of miR-3188-loaded exosomes inhibited xenograft tumor growth in nude mice. **b** Immunohistochemical analysis of BCL2 expression, Ki-67 staining, Caspase3 expression and TUNEL assay in the xenografts (Scale bar: main = 50 μm; insert = 20 μm). (NC, negative control. ns, no significant difference, *p < 0.05, **p < 0.01, ***p < 0.001, ****p < 0.0001)

Finally, to determine whether the exosomes loaded with miR-3188 had an anti-tumor effect in vivo, we administered miR-3188-loaded exosomes (Additional file 4: Figure S10c) to BALB/C nude mice bearing CAL 27 xenografts. The tumor volumes were significantly lower in the group receiving intra-tumor injections of miR-3188-loaded exosomes than in the group treated with miR-NC-loaded exosomes (Fig. 7a). In addition, treatment with exosomes loaded with miR-3188 decreased the expression of BCL2 and Ki-67 and increased the expression of the apoptotic marker caspase-3. Moreover, the TUNEL assays indicated improved levels of apoptosis (Fig. 7b). Taken together, these findings suggest that miR-3188-loaded exosomes had an anti-tumor effect in the xenograft model.

### The miR-3188 level in tumor tissues and plasma correlates with tumor progression in HNC patients

We analyzed the correlations between miR-3188 expression levels in tumor tissues and the clinicopathological parameters of HNC patients. As shown in Table 1, lower miR-3188 expression was significantly correlated with larger tumor size (greater than 4 cm in diameter). In addition, the results showed that BCL2 was overexpressed in larger tumors (Table 2). Next, we measured miR-3188 expression in another cohort of 64 paired HNC samples using real-time PCR. Consistent with the above data, miR-3188 levels were lower in the tumor tissues than in the adjacent normal tissues (Additional file 4: Figure S12a). Furthermore, Kaplan-Meier analysis was performed to analyze the overall survival of HNC patients and the result showed that HNC patients with lower miR-3188 expression had poorer survival (*p* = 0.0040, Additional file 4: Figure S12b).

**Table 1:** Relationship between miR-3188 level and clinicopathologic features (*N* = 115)

Characteristics	No. of Patients		miR-3188 ΔCt^a^ Mean ± SD	*P* value
	No.	%		
Age (years)				
≥ 60	53	46.1	7.894 ± 2.510	0.1616
< 60	62	53.9	8.441 ± 1.848	
Gender				
Male	82	71.3	8.352 ± 2.233	0.1503
Female	33	28.7	7.784 ± 2.030	
Smoking history				
Nonsmoker	76	66.1	8.142 ± 2.378	0.8366
Smoker	39	33.9	8.280 ± 1.777	
Alcohol history				
Nondrinker	82	71.3	8.156 ± 2.279	0.6264
Drinker	33	28.7	8.271 ± 1.964	
Tumor size (cm)				
≤ 4	89	77.4	8.602 ± 1.796	0.0045
> 4	26	22.6	6.776 ± 2.783	
Lymph node metastasis				
Non-metastasis	63	54.8	8.062 ± 2.100	0.7506
Metastasis	52	45.2	8.343 ± 2.296	
TNM stage				
I	69	60	8.668 ± 1.632	0.0160
II	41	35.7	8.182 ± 1.605	
III	5	4.3	7.347 ± 2.408	
Pathological Differentiation				
Well	78	67.8	7.955 ± 2.301	0.2020
Moderately/poorly	37	32.2	8.683 ± 1.852	
Disease Site				
Tongue	42	36.5	8.034 ± 2.020	0.9280
Gingival	36	31.3	8.196 ± 2.110	
Cheek	18	15.7	7.831 ± 2.442	
Floor of Mouth	12	10.4	8.702 ± 2.417	
Oropharynx	7	6.1	9.124 ± 2.684	

*Abbreviations*: *SD* Standard deviation, *TNM stage* Tumor-lymph node-metastasis stage

^a^△Ct indicates the difference in the cycle number at which a sample’s fluorescent signal passes a given threshold above baseline (Ct) derived from a specific gene compared with that of β-actin in tumor tissues

**Table 2:** Relationship between BCL2 level and clinicopathologic features (*N* = 115)

Characteristics	No. of Patients		BCL2 ΔCt^a^ Mean ± SD	*P* value
	No.	%		
Age (years)				
≥ 60	53	46.1	5.475 ± 3.733	0.5450
< 60	62	53.9	4.974 ± 3.584	
Gender				
Male	82	71.3	5.108 ± 3.992	0.7880
Female	33	28.7	5.445 ± 2.631	
Smoking history				
Nonsmoker	76	66.1	5.313 ± 3.353	0.8890
Smoker	39	33.9	4.994 ± 4.196	
Alcohol history				
Nondrinker	82	71.3	5.404 ± 3.420	0.6564
Drinker	33	28.7	4.710 ± 4.196	
Tumor size (cm)				
≤ 4	89	77.4	4.682 ± 3.567	0.0168
> 4	26	22.6	6.994 ± 3.393	
Lymph node metastasis				
Non-metastasis	63	54.8	5.096 ± 3.713	0.7979
Metastasis	52	45.2	5.337 ± 3.594	
TNM stage				
I	69	60.0	5.835 ± 2.991	0.3083
II	41	35.7	4.282 ± 3.924	
III	5	4.3	5.972 ± 3.650	
Pathological Differentiation				
Well	78	67.8	4.834 ± 3.916	0.1040
Moderately/poorly	37	32.2	5.986 ± 2.891	
Disease Site				
Tongue	42	36.5	4.654 ± 4.016	0.1607
Gingival	36	31.3	6.024 ± 3.281	
Cheek	18	15.7	5.948 ± 4.205	
Floor of Mouth	12	10.4	4.743 ± 2.077	
Oropharynx	7	6.1	3.180 ± 2.684	

*Abbreviations*: *SD* Standard deviation, *TNM stage* Tumor-lymph node-metastasis stage

^a^△Ct indicates the difference in the cycle number at which a sample’s fluorescent signal passes a given threshold above baseline (Ct) derived from a specific gene compared with that of β-actin in tumor tissues

We next explored whether circulating miR-3188 levels could predict the diagnosis of HNC. Plasma miR-3188 levels were decreased in HNC patients (Additional file 4: Figure S12c) and increased after surgery compared to those pre-operation (Additional file 4: Figure S12d). A receiver operating characteristic (ROC) curve was used to prove that plasma miR-3188 levels could discriminate HNC patients from healthy individuals (Additional file 4: Figure S12e). Moreover, we analyzed the correlations between plasma miR-3188 expression level and the clinicopathological parameters of HNC patients. The data showed that lower miR-3188 expression was significantly correlated with larger tumor sizes (greater than 4 cm in diameter) and advanced TNM stage (Additional file 4: Figure S12 f, g). Besides, other clinicopathological parameters included age, gender, smoking history, alcohol history, lymph node metastasis, pathological differentiation and disease site were not significantly associated with miR-3188 levels (Additional file 4: Figure S13 a-g). In conclusion, the results indicated that miR-3188 might serve as a diagnostic factor for HNC patients.

## Discussion

Although the gradual accumulation of genetic lesions creates the initial “spark” necessary for disease initiation, it is widely acknowledged that the TME plays a critical role at every stage of malignant progression [5]. As the predominant component of the TME, CAFs are responsible for a series of malignant transformations, including tumor proliferation, metastasis, invasion and other critical oncological behaviors. MiRNAs play a regulatory role in not only cancer cells but also CAF-mediated tumor progression [30, 31]. Increasing evidence has revealed that the dysregulation of miRNAs in CAFs or CAF-derived exosomes functionally influences the crosstalk between tumor cells and the TME. In the present study, we initially compared the miRNA profiles of 3 paired CAFs and NFs using miRNA-seq. Consistent with a previous report, the data indicate that a set of miRNAs is aberrantly expressed in CAFs compared with those in NFs. Moreover, miR-3188 was found to be the most downregulated miRNA in CAF-derived exosomes by miRNA array analysis. Subsequent experiments verified the reduced miR-3188 expression levels in CAF-derived exosomes, and our results also suggested that the transmission of miR-3188 from fibroblasts to tumor cells was mainly dependent on exosomes. Further experiments demonstrated that the ectopic expression of miR-3188 could suppress tumor cell growth and induce apoptosis by targeting BCL2 both in vitro and in vivo. The findings of our study revealed the impact of the loss of specific miRNAs in CAF-derived exosomes on HNC cells. In addition, the downregulation of miR-3188 in the tissue and plasma of HNC patients indicates that miR-3188 could act as a biomarker for HNC prognosis. However, the miRNA array data indicated that a set of miRNAs is aberrantly expressed in CAF-derived exosomes, and further studies are needed to elucidate the role of other differentially expressed miRNAs in those exosomes.

In addition, our data corroborate that CAFs and NFs do have different characteristics and functions. We demonstrated that although weaker than CAFs, NFs could facilitate the proliferation, migration and invasion of tumor cells. Furthermore, treatment with fibroblast-secreted exosomes had effects similar to co-culture with fibroblasts, suggesting the critical role of the biological functions of exosomes. Many studies have confirmed the contribution of miRNAs in CAF-derived exosomes to the regulation of tumor cell behavior. However, there is little evidence about whether the loss of exosomal miRNA could regulate tumor cell behavior. The present study demonstrates that not only the overexpression but also the loss of certain miRNAs in CAF-derived exosomes could facilitate the progression of tumors.

**Fig. 8:**
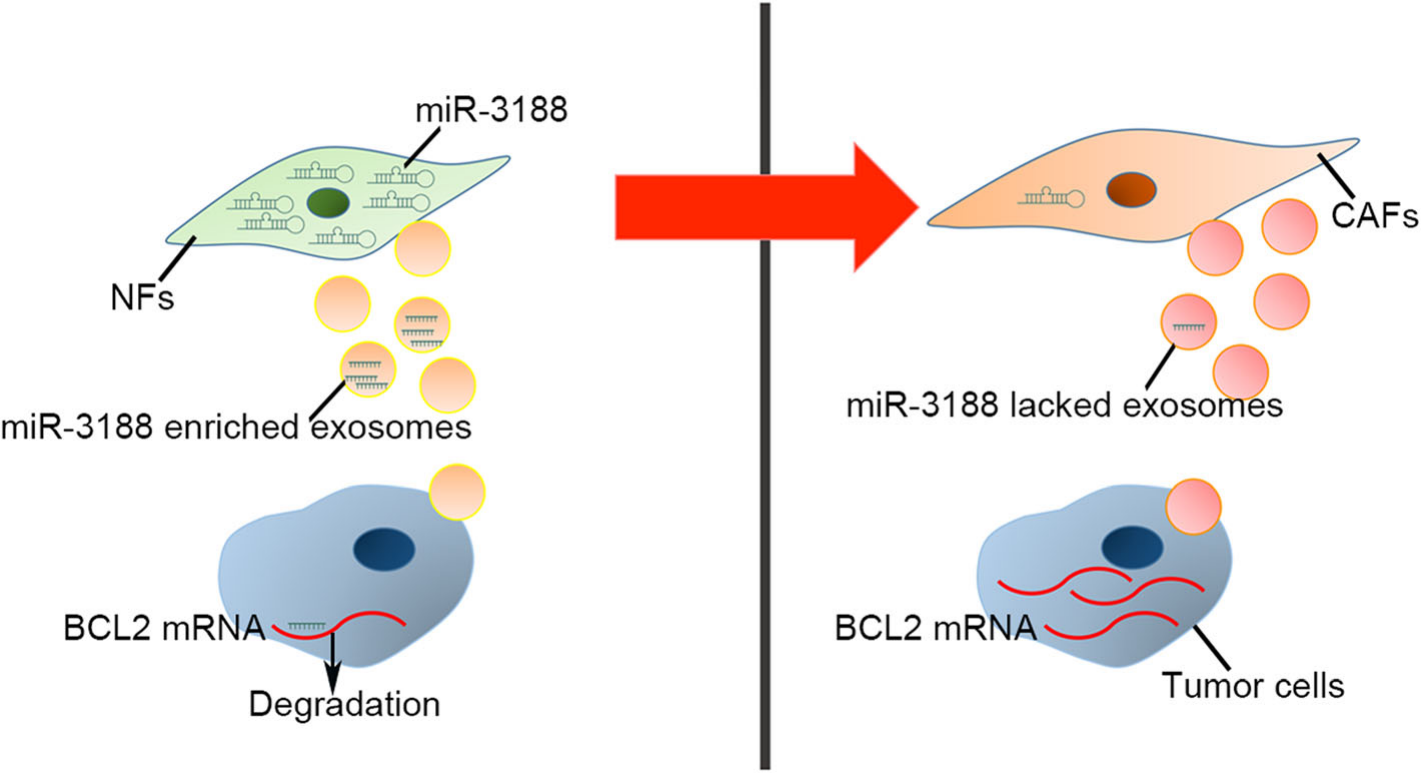
A schematic diagram of proposed mechanism. The conversion from NFs to CAFs causes a reduction of miR-3188. Subsequently, the low level of miR-3188 in the CAF-derived exosomes contributes to the derepression of BCL2, which promotes the aggression of HNC cells

MiR-3188 has been reported to be dysregulated in several tumors [32–34], but information on its biological role, especially in HNC, is limited. Zhao et al. demonstrated that reduced miR-3188 was an unfavorable factor in nasopharyngeal carcinoma (NPC) clinical samples and that miR-3188 directly targeted mTOR and mediated NPC cell growth, tumorigenesis and chemotherapy resistance [34]. However, another study in hepatitis B virus-related HCC reached a conflicting conclusion. That study found that miR-3188 was markedly overexpressed in HCC tissues and that miR-3188 knockout suppressed cell growth, migration, invasion and tumorigenesis [32]. The opposite function of miR-3188 may be due to the different genetic backgrounds and origins of these cell types. Here, our data verified the reduced expression levels of miR-3188 in both tissues and plasma from HNC patients. In addition, the ectopic expression of miR-3188 could suppress tumor cell growth both in vitro and in vivo by inhibiting the antiapoptotic regulator BCL2. Therefore, we proposed a model in which the conversion from NFs to CAFs causes a reduction in miR-3188. Subsequently, the low levels of miR-3188 in the exosomes derived from fibroblasts induced more aggressive characteristics in tumor cells (Fig. 8).

BCL2 is an anti-apoptosis member of the BCL2 family, and this protein inhibits apoptosis by preventing activators (BID, BIM, PUMA) from engaging the effectors BAX and BAK [35]. BCL2 overexpression is observed in many tumors, so this anti-apoptotic protein is now well established as a validated, high-value cancer target [36]. As a typical BH3 mimetics, venetoclax was recently approved by Food and Drug Administration for the treatment of relapsed chronic lymphocytic leukemia with 17p deletion. Although venetoclax is limited to the treatment of solid tumor, other new BCL2 specific inhibitors are now underway. For example, Bingshe Han et al. identified a small molecule Bcl2-BH4 domain-antagonist (BDA-366) that binds BH4 with high affinity and selectivity. BDA-366-Bcl2 binding could convert BCL2 from a survival to a cell death inducer. In addition, BDA-366 suppresses growth of lung cancer xenografts derived from cell lines and patient (PDX model) without significant normal tissue toxicity at effective doses [37]. Combination with other chemotherapeutic agents is another potent strategy for the application of BCL2 inhibitors. It is reported that combination with venetoclax could enhance the anti-cancer effect of tamoxifen in patients with ER and BCL2-positive metastatic breast cancer and a clinical study is undergoing [38]. There have been reports indicating that BCL2 can predict an unfavorable prognosis and negative response to chemotherapy and radiotherapy for HNC [39–41]. Our present study revealed BCL2 overexpression in HNC samples. Moreover, the analysis of the relationship between BCL2 expression and the characteristics of HNC patients indicated that BCL2 might influence HNC cell growth. In addition, it was reported that BCL2 could form a complex with actin and gelsolin that functions to increase actin polymerization and suppress the cell adhesion process [42]. These findings indicate that BCL2 might affect the motility of tumor cells. However, in our study, we observed no significant difference in migration and invasion after the alteration of BCL2 in HN4 and HN30 cells. These conflicting results may be associated with the different genetic backgrounds and origins of these cell types, and further experiments are needed to elucidate the migration and invasion effects of BCL2. Interestingly, we found that silencing BCL2 downregulated cyclin D1, thus triggering cell cycle arrest. However, the underlying mechanism needs to be further investigated in the future.

A series of reports demonstrated that exosomes are expected to be effective therapeutic reagents for various diseases, including cancers. The earliest research presented exosome vaccinations as a possible application. Autologous dendritic cell (DC)-derived exosomes from metastatic melanoma patients were purified and loaded with melanoma antigen-encoding gene 3 (MAGE3) antigenic peptides. Then, the loaded exosomes were re-injected into patients. The treatment restored NKG2D expression in NK cells and CD8^+^ cells in some patients, which indicated the improvement of immunoactivity [43]. Several other trials have shown that exosome vaccinations could generate an immune response against tumors, thus indicating a promising use for exosomes [44, 45]. Another exciting application of exosomes for therapeutic development is their use as delivery vehicles for non-native therapeutics, including nucleic acids, proteins and small molecule drugs [28]. Li et al. demonstrated that fibroblast-derived exosomes loaded with miR-195 can be administered in a rat model of cholangiocarcinoma; these exosomes concentrated within the tumor, decreased the tumor size and improved survival in the treated rats [46]. In addition, Sushrut et al. engineered exosomes derived from normal fibroblast-like mesenchymal cells to carry siRNA or shRNA specific to oncogenic KRAS^G12D^; these exosomes were then used to treat multiple mouse models of pancreatic cancer. Excitingly, the exosomes targeting KRAS suppressed tumor development and significantly increased mouse overall survival [47]. In this study, we found that the utility of an exosome inhibitor could affect the crosstalk between tumor cells and fibroblasts in vivo. More importantly, our data revealed that the miR-3188-loaded exosomes effectively suppressed the growth of HNC xenografts. These data, along with additional reports, shed light on the potential therapeutic application of exosomes.

An increasing number of studies have reported the diagnostic potential of circulating miRNAs, and it has recently become clear that bloodborne miRNAs are protected mainly by packaging in exosomes [28]. Therefore, we preliminarily researched plasma miRNA expression in HNC patients in this study. Our results revealed that plasma miR-3188 could serve as a potential biomarker for the diagnosis of HNC. Nevertheless, stricter inclusion criteria should be established, and a larger cohort should be used to prove the true diagnostic efficiency of miR-3188 in future studies.

## Conclusions

In conclusion, our findings for the first time confirmed that CAF-derived exosomes contain lower levels of miR-3188 than NFs. Importantly, miR-3188 could serve as a signal mediator controlling the interaction between CAFs and tumor cells. The loss of miR-3188 in exosomes promotes the malignant phenotypes of HNC cells through the derepression of BCL2. In addition, the in vivo study indicated the potential therapeutic value of exosomal miR-3188 for inhibiting HNC growth. Furthermore, analysis of the clinical data showed the potent diagnostic value of miR-3188 for HNC patients.

## Additional files

Additional file 1: **Table S1.** Primary antibodies used for Western blotting, Immunohistochemistry, and Immunofluorescence. (DOC 42 kb)

Additional file 2: **Table S2.** The primers used in this study. (DOC 14 kb)

Additional file 3: **Table S3.** The Sequences used in this study. (DOC 61 kb)

Additional file 4: **Figure S1.** Co-culture with CAFs prompts the malignant phenotypes of HNC cells. **Figure S2.** Identification of exosomes purified from CAFs and NFs. **Figure S3.** Treatment with fibroblast-exosomes prompts the malignant phenotypes of HNC cells. **Figure S4.** MiR-3188 expression in HNC cells after transfected with miR-3188 mimics or inhibitor. **Figure S5.** miR-3188 inhibits the growth of HN30 cells and facilitates the apoptosis of HN30 cells in vitro*.* **Figure S6.** Functional analyses of the effects of miR-3188 on HNC cell migration and invasion. **Figure S7.** BCL2 facilitates the growth of HN30 cells and inhibits the apoptosis of HN30 cells in vitro*.* **Figure S8.** The expression of Cyclin D1 in HNC cells after BCL2 knockdown or overexpression. **Figure S9.** BCL2 expression was recovered by the transfection of BCL2 expression vector in miR-3188-expressing HNC cells**. Figure S10.** MiR-3188 expression in CAL27, NFs and exosomes after transfection of miR-3188 mimics or inhibitor. **Figure S11.** MiR-3188 and BCL2 mRNA expression in xenografts. **Figure S12.** MiR-3188 expression in HNC tissues and plasma of HNC patients. **Figure S13.** The correlation of plasma miR-3188 expression and clinicopathological characteristics of HNC patients. (PPTX 22445 kb)

Additional file 5: **Table S4.** The differentially expressed miRNA in 3 pairs of NFs and CAFs. (DOC 234 kb)

## Abbreviations

BCL2: B-cell lymphoma 2
CAFs: Cancer associated fibroblasts
CM: Conditioned medium
EdU: 5-ethynyl-2′-deoxyuridine
GFP: Green fluorescent protein
HNC: Head and neck cancer
miRNA: microRNA
mTOR: The mammalian target of rapamycin
MTT: 3-(4,5-dimethyl-2-thiazolyl)-2,5-diphenyl-2-H-tetrazolium bromide, Thiazolyl Blue Tetrazolium Bromide
NC: Negative control
NFs: Normal fibroblasts
NTA: Nanoparticle tracking analysis
PBX3: Pre-B cell leukemia transcription factors 3
PCR: Polymerase chain reaction
PVDF: Polyvinylidene fluoride
ROC: Receiver operating characteristic
TME: Tumor microenvironment
TUNEL: Terminal deoxynucleotidyl transferase dUTP nick and labeling
