# Supplementary material for: Loss of exosomal miR-3188 in cancer-associated fibroblasts contributes to HNC progression
Source: J Exp Clin Cancer Res. 2019 Apr 8;38:151. doi: 10.1186/s13046-019-1144-9 (PMC6454737; doi:10.1186/s13046-019-1144-9)
Supplement: Supplementary file 1 — Table S1. Primary antibodies used for Western blotting, Immunohistochemistry, and Immunofluorescence. (DOC 42 kb) [file 13046_2019_1144_MOESM1_ESM.doc]

**Supplementary Table 1.** Primary antibodies used for Western blotting, Immunohistochemistry, and Immunofluorescence

| **Usage** | **Name of antibody** | **Company** | **Dilution** |
| --- | --- | --- | --- |
| **Western blotting** | FAP | Abcam, USA | 1:1,000 |
|  | α-SMA | Abcam, USA | 1:300 |
|  | FSP1 | Abcam, USA | 1:1,000 |
|  | GAPDH | Proteintech, USA | 1:1,000 |
|  | Alix | Invitrogen, USA | 1:1,000 |
|  | HSP90 | Boster, China | 1:1,000 |
|  | CD63  [EPR5702] | Abcam, USA | 1:1,000 |
|  | CD9 | Proteintech, USA | 1:1,000 |
|  | CD81 | Proteintech, USA | 1:1,000 |
|  | GM130 | Abcam, USA | 1:1,000 |
|  | BCL2 | Proteintech, USA | 1:1,000 |
|  | PARP  [46D11] | CST, USA | 1:1,000 |
|  | Cyclin D1 | Proteintech, USA | 1:1,000 |
|  | BAX | Proteintech, USA | 1:1,000 |
|  | Cleaved Caspase 3 | Proteintech, USA | 1:1,000 |
|  | β-Tubulin | Sigma-Aldrich | 1:5,000 |
| **Immuno-**  **histochemistry** | BCL2 | Proteintech, USA | 1:200 |
|  | Ki67  [20Raj1] | eBioscience, USA | 1:200 |
| **Immuno-**  **fluorescence** | FAP | Abcam, USA | 1:200 |
|  | FSP | Abcam, USA | 1:200 |
|  | α-SMA | Abcam, USA | 1:50 |
