# Supplementary material for: Loss of exosomal miR-3188 in cancer-associated fibroblasts contributes to HNC progression
Source: J Exp Clin Cancer Res. 2019 Apr 8;38:151. doi: 10.1186/s13046-019-1144-9 (PMC6454737; doi:10.1186/s13046-019-1144-9)
Supplement: Supplementary file 2 — Table S2. The primers used in this study. (DOC 14 kb) [file 13046_2019_1144_MOESM2_ESM.doc]

**Supplementary Table 2: The primers used in this study**

| **Primers name** |  | **Sequence (5'- 3')** |
| --- | --- | --- |
| BCL2 | Forward | GGTGGGGTCATGTGTGTGG |
|  | Reverse | CGGTTCAGGTACTCAGTCATCC |
| DHCR24 | Forward | GCCGCTCTCGCTTATCTTCG |
|  | Reverse | GTCTTGCTACCCTGCTCCTT |
| ARHGEF2 | Forward | CAGGCATGACCATGTGCTATG |
|  | Reverse | TTTACAGCGGTTGTGGATAGTC |
| MAPK1 | Forward | TCACACAGGGTTCCTGACAGA |
|  | Reverse | ATGCAGCCTACAGACCAAATATC |
| MNAT1 | Forward | GGTTGCCCTCGGTGTAAGAC |
|  | Reverse | AGTTGCTCTTTCTGAGTGGAGT |
| CDKN1A | Forward | TGTCCGTCAGAACCCATGC |
|  | Reverse | AAAGTCGAAGTTCCATCGCTC |
| β-Actin | Forward | CATGTACGTTGCTATCCAGGC |
|  | Reverse | CTCCTTAATGTCACGCACGAT |
| miR-3188 |  | AGAGGCTTTGTGCGGATACGGG |
| U6 |  | CTCGCTTCGGCAGCACATATA |
