# Supplementary material for: Loss of exosomal miR-3188 in cancer-associated fibroblasts contributes to HNC progression
Source: J Exp Clin Cancer Res. 2019 Apr 8;38:151. doi: 10.1186/s13046-019-1144-9 (PMC6454737; doi:10.1186/s13046-019-1144-9)
Supplement: Supplementary file 3 — Table S3. The Sequences used in this study. (DOC 61 kb) [file 13046_2019_1144_MOESM3_ESM.doc]

**Supplementary Table 3: The Sequences used in this study.**

| **Gene** |  | **Sequence** |
| --- | --- | --- |
| siBCL2 | Sense | 5' UGUGGAUGACUGAGUACCUGAdTdT 3' |
|  | Antisense | 3'dTdTACACCUACUGACUCAUGGACU5' |
| siNC | Sense | 5'UUCUCCGAACGUGUCACGUdTdT3' |
|  | Antisense | 3'dTdTAAGAGGCUUGCACAGUGCA5' |
| miR-3188 mimics | Sense | 5'AGAGGCUUUGUGCGGAUACGGGG3' |
|  | Antisense | 3'UCUCCGAAACACGCCUAUGCCCC5' |
| mimics NC | Sense | 5'UCACAACCUCCUAGAAAGAGUAGA3' |
|  | Antisense | 3'UCUACUCUUUCUAGGAGGUUGUGA5' |
| miR-3188 inhibitor |  | 5'CCCCGUAUCCGCACAAAGCCUCU3' |
| Inhibitor NC |  | 5'UCUACUCUUUCUAGGAGGUUGUGA3' |
