# Supplementary material for: Loss of exosomal miR-3188 in cancer-associated fibroblasts contributes to HNC progression
Source: J Exp Clin Cancer Res. 2019 Apr 8;38:151. doi: 10.1186/s13046-019-1144-9 (PMC6454737; doi:10.1186/s13046-019-1144-9)
Supplement: Supplementary file 4 — Figure S1. Co-culture with CAFs prompts the malignant phenotypes of HNC cells. Figure S2. Identification of exosomes purified from CAFs and NFs. Figure S3. Treatment with fibroblast-exosomes prompts the malignant phenotypes of HNC cells. Figure S4. MiR-3188 expression in HNC cells after transfected with miR-3188 mimics or inhibitor. Figure S5. miR-3188 inhibits the growth of HN30 cells and facilitates the apoptosis of HN30 cells in vitro. Figure S6. Functional analyses of the effects of miR-3188 on HNC cell migration and invasion. Figure S7. BCL2 facilitates the growth of HN30 cells and inhibits the apoptosis of HN30 cells in vitro. Figure S8. The expression of Cyclin D1 in HNC cells after BCL2 knockdown or overexpression. Figure S9. BCL2 expression was recovered by the transfection of BCL2 expression vector in miR-3188-expressing HNC cells. Figure S10. MiR-3188 expression in CAL27, NFs and exosomes after transfection of miR-3188 mimics or inhibitor. Figure S11. MiR-3188 and BCL2 mRNA expression in xenografts. Figure S12. MiR-3188 expression in HNC tissues and plasma of HNC patients. Figure S13. The correlation of plasma miR-3188 expression and clinicopathological characteristics of HNC patients. (PPTX 22445 kb) [file 13046_2019_1144_MOESM4_ESM.pptx]

## Slide 1
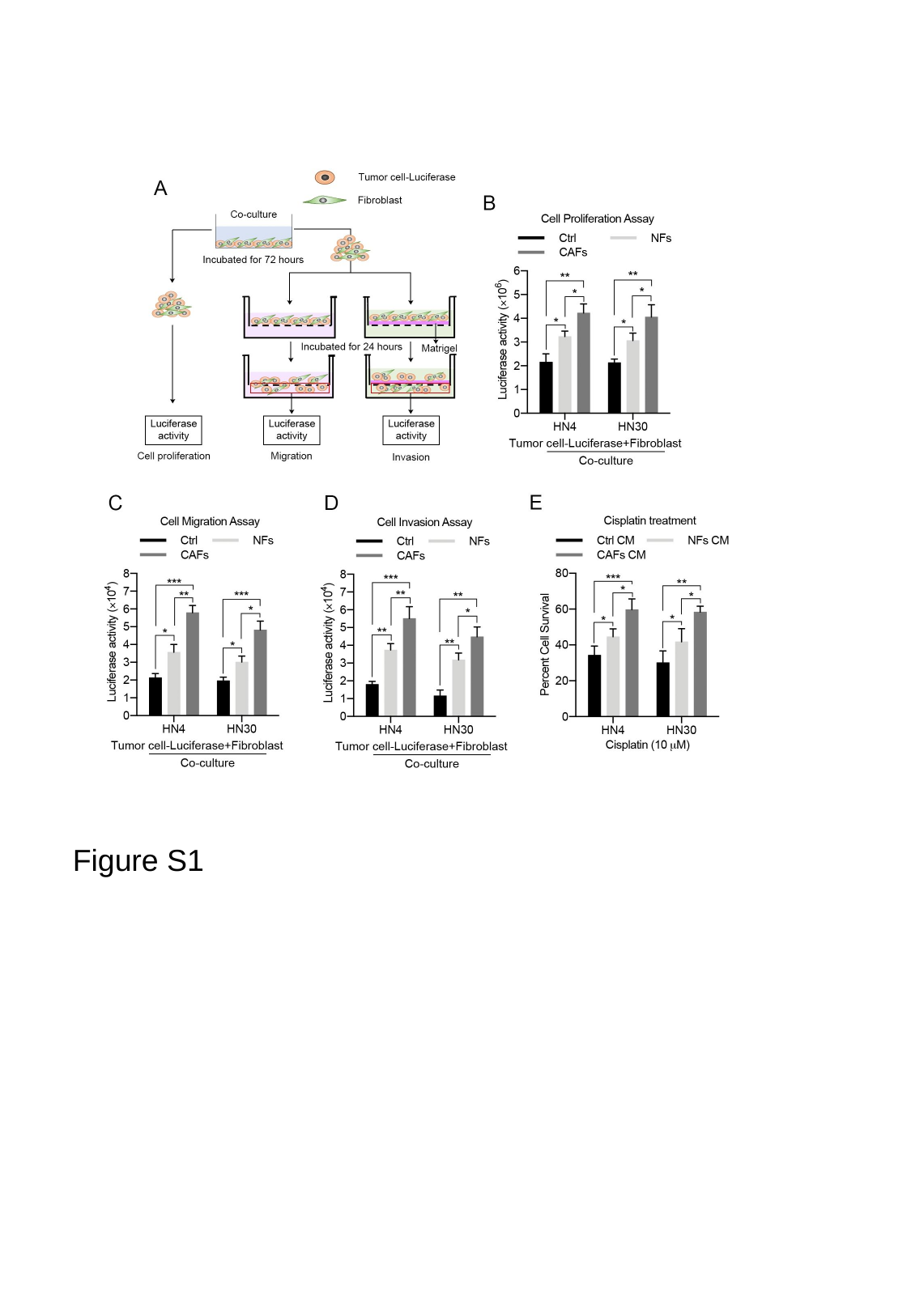

Figure S1

## Slide 2
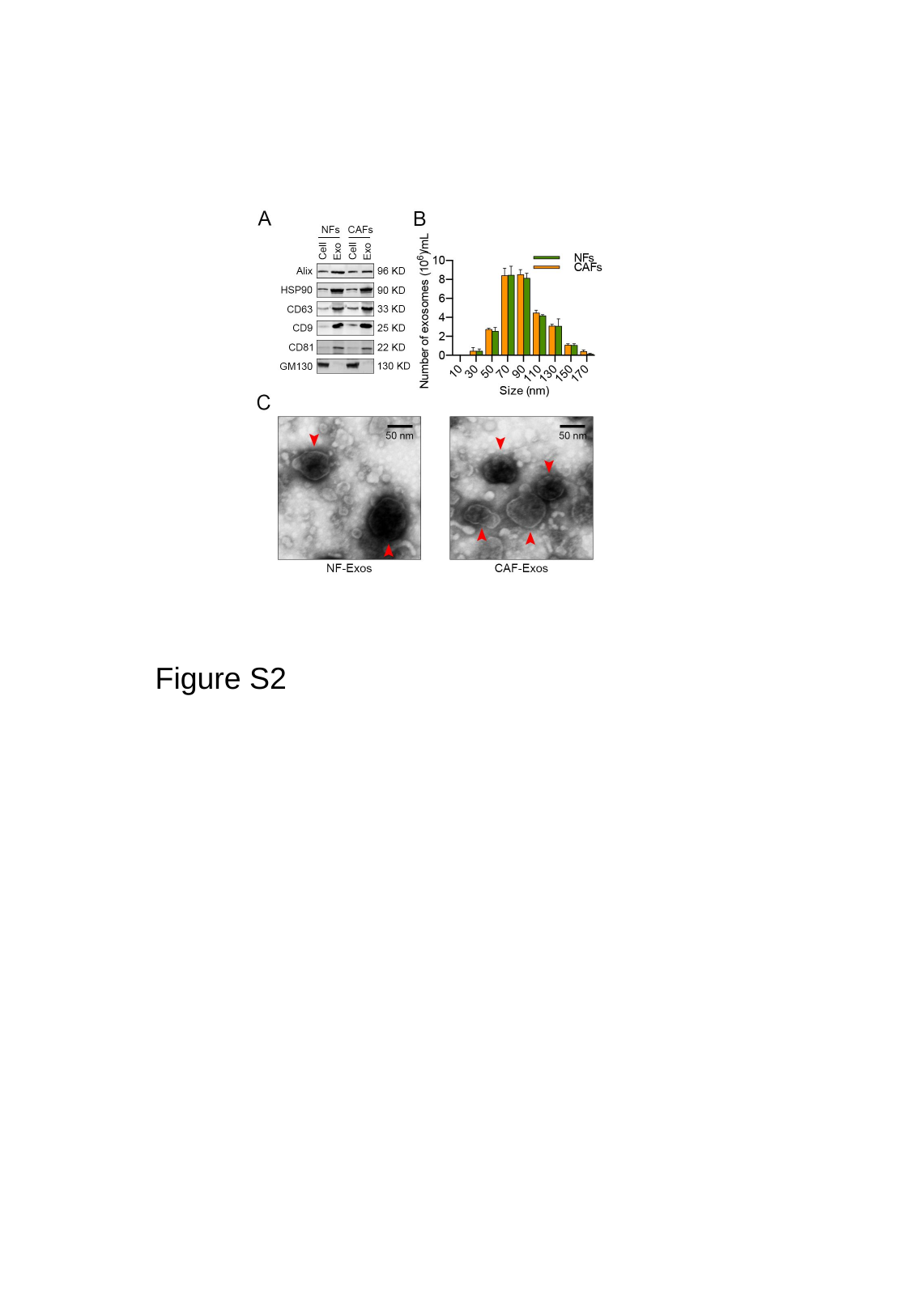

Figure S2

## Slide 3
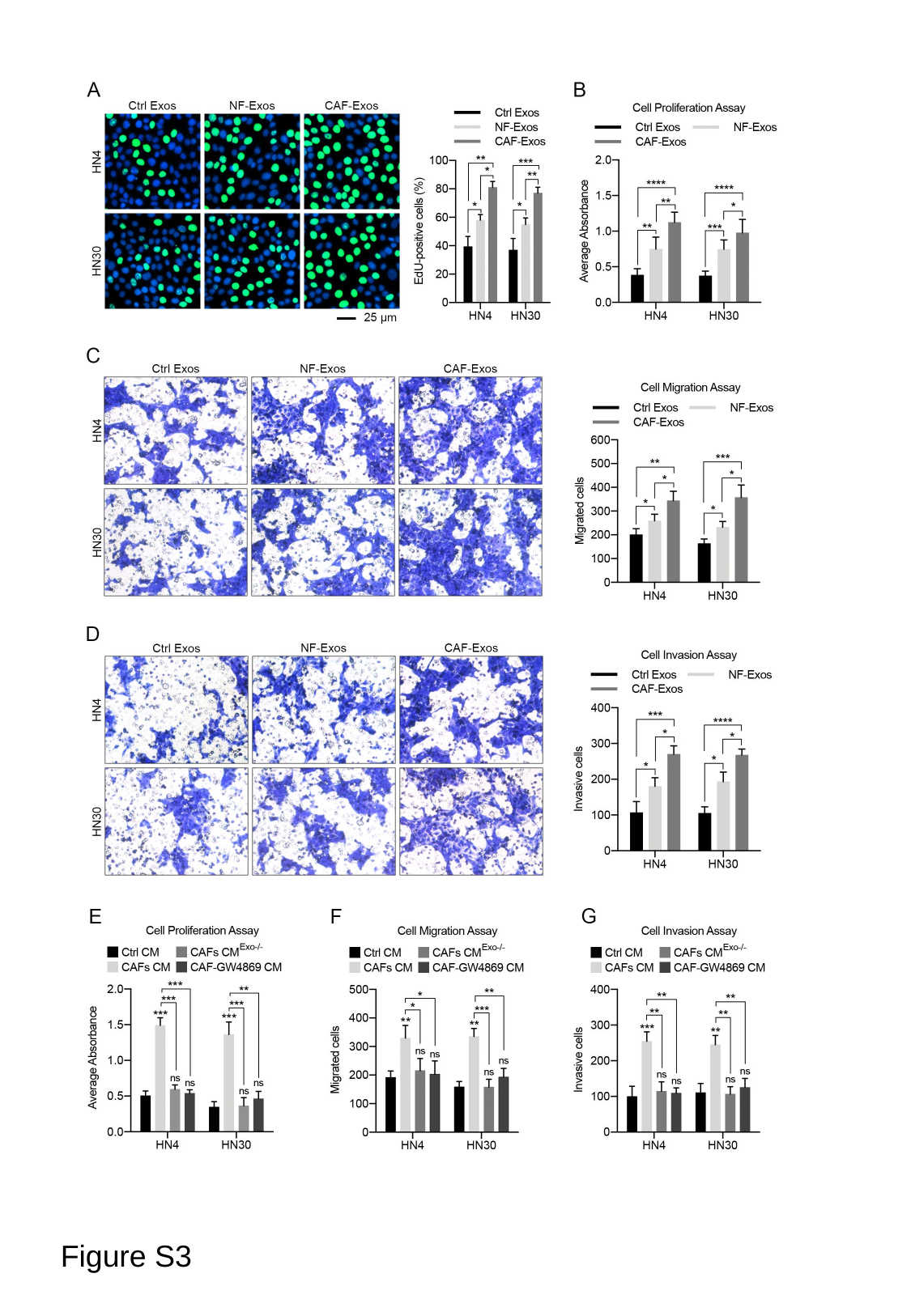

Figure S3

## Slide 4
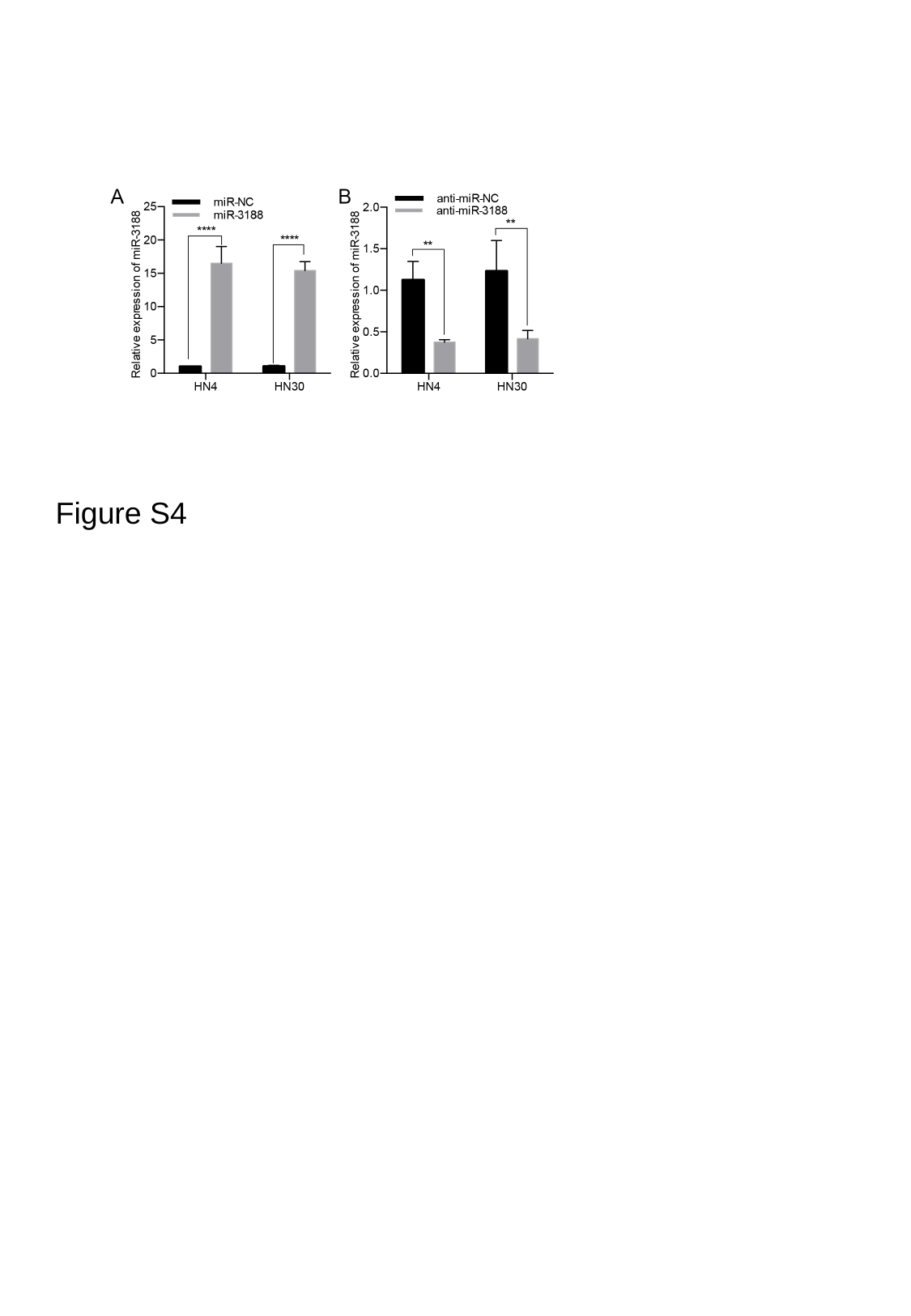

Figure S4

## Slide 5
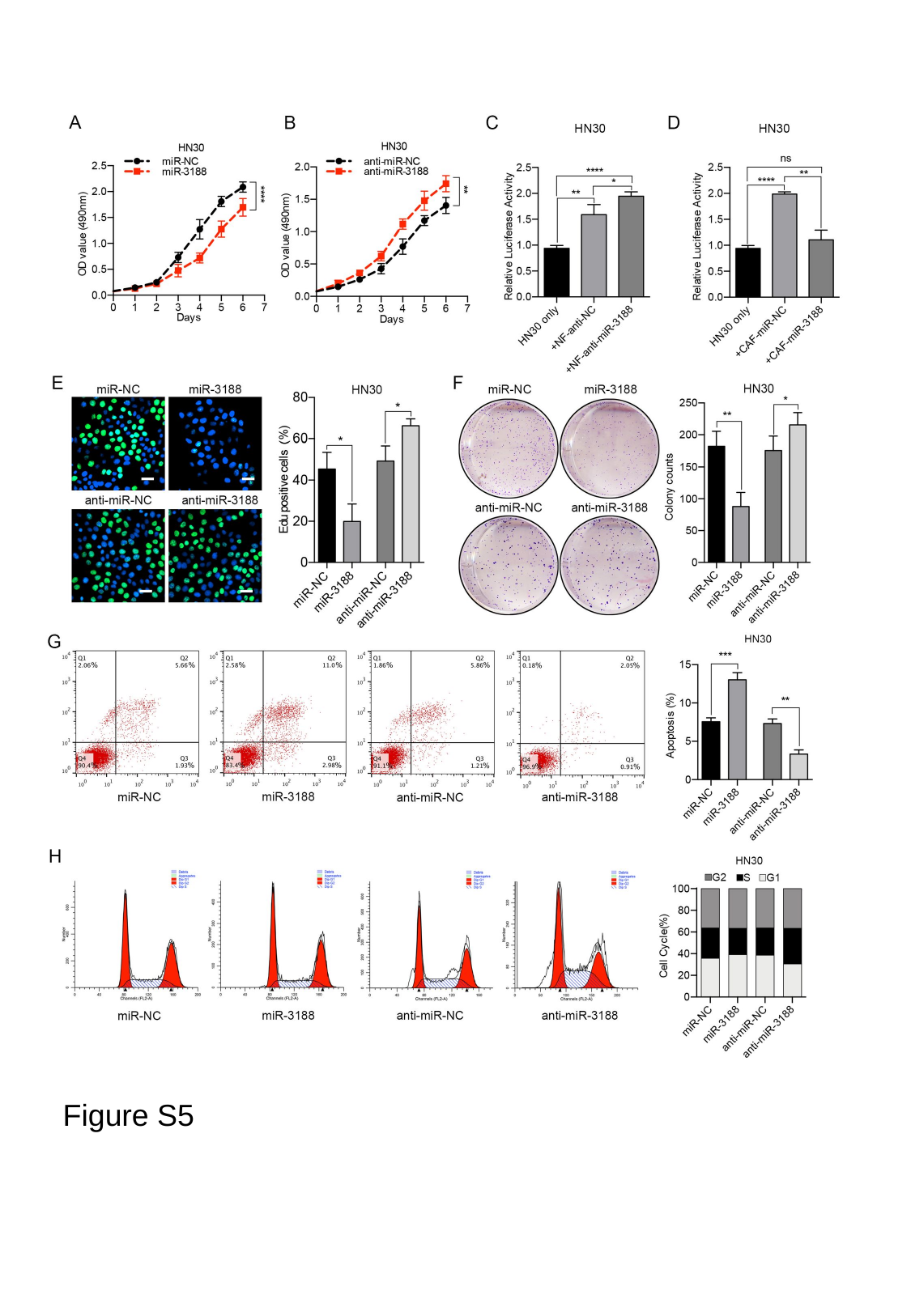

Figure S5

## Slide 6
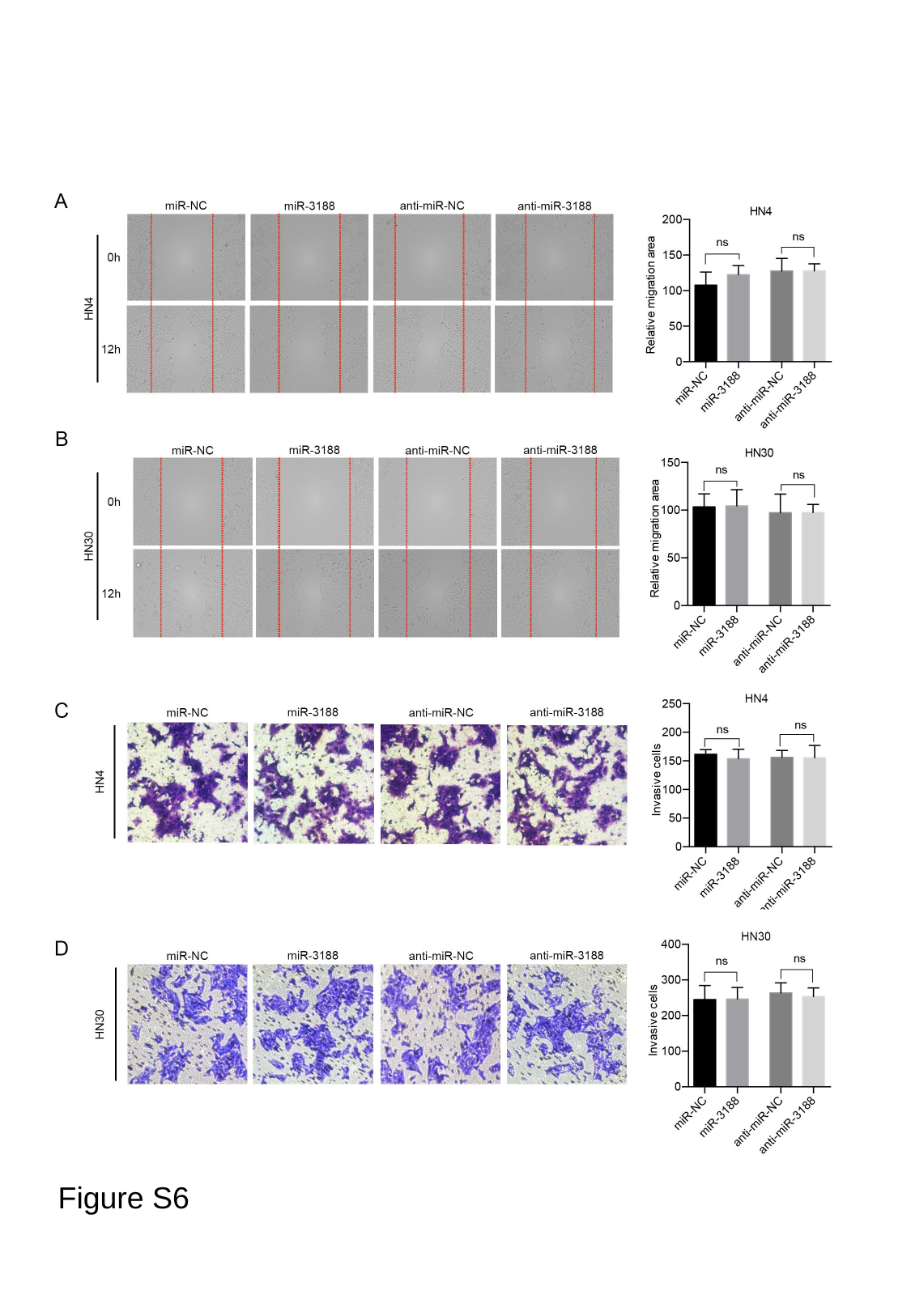

Figure S6

## Slide 7
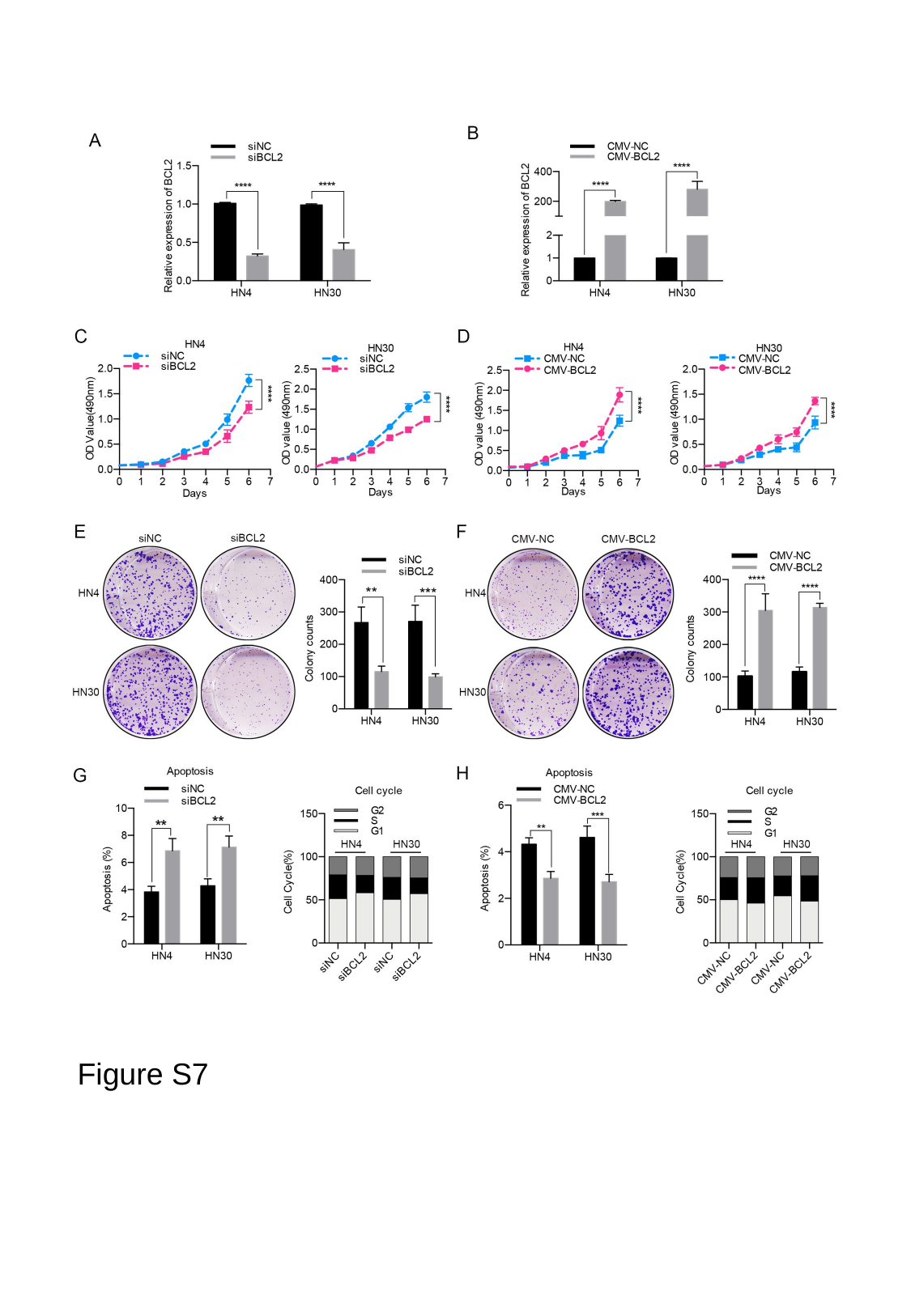

Figure S7

## Slide 8
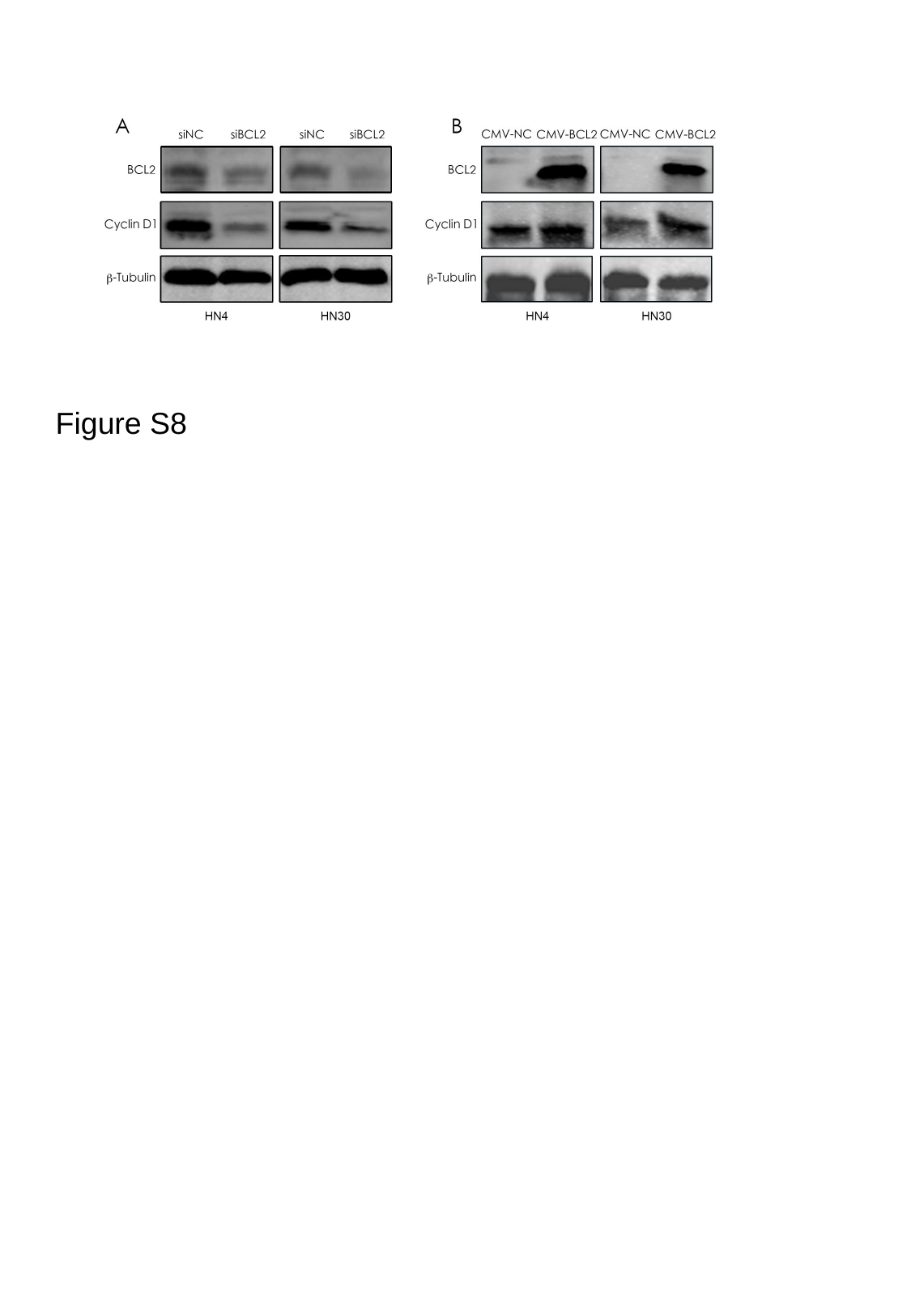

Figure S8

## Slide 9
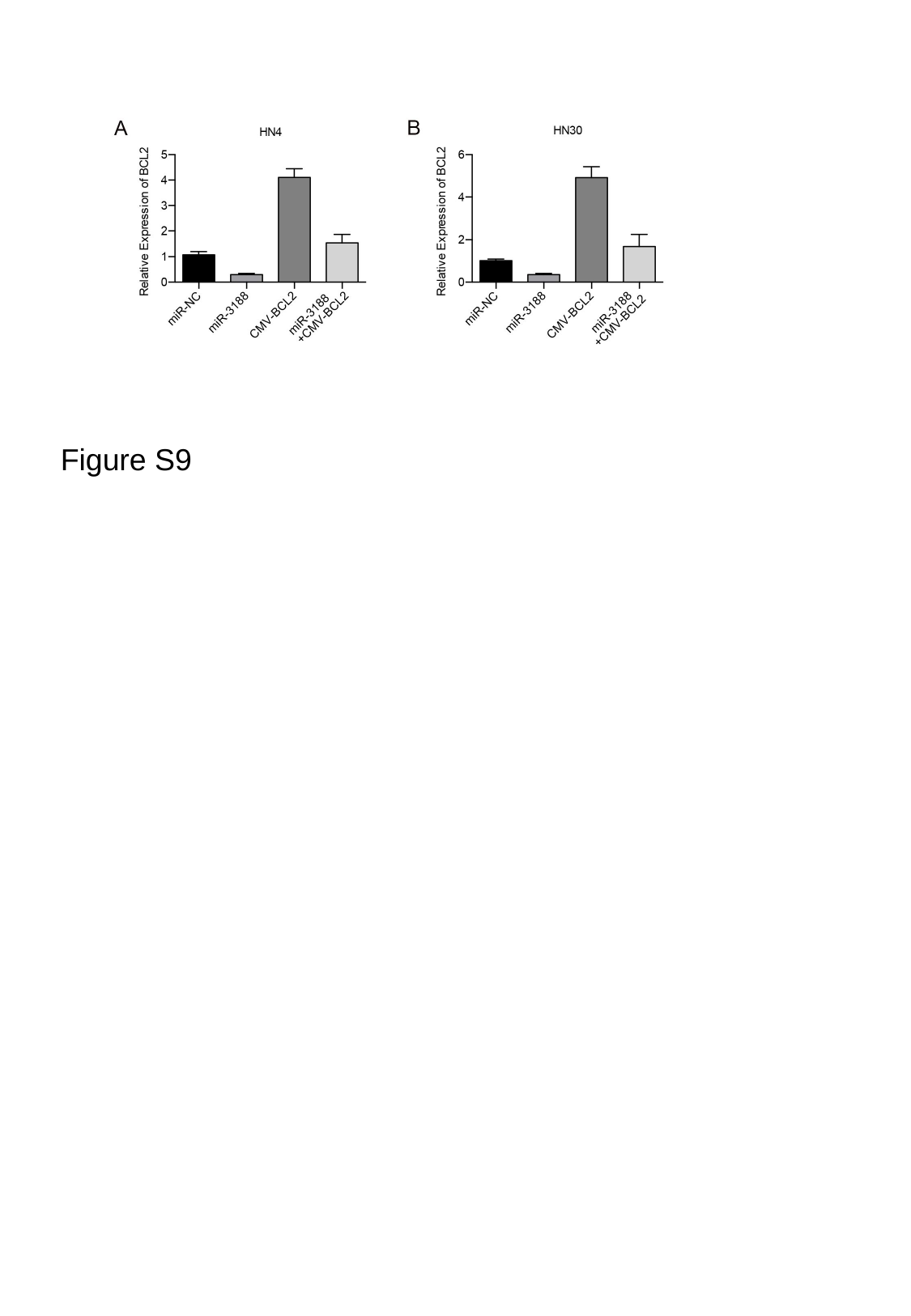

Figure S9

## Slide 10
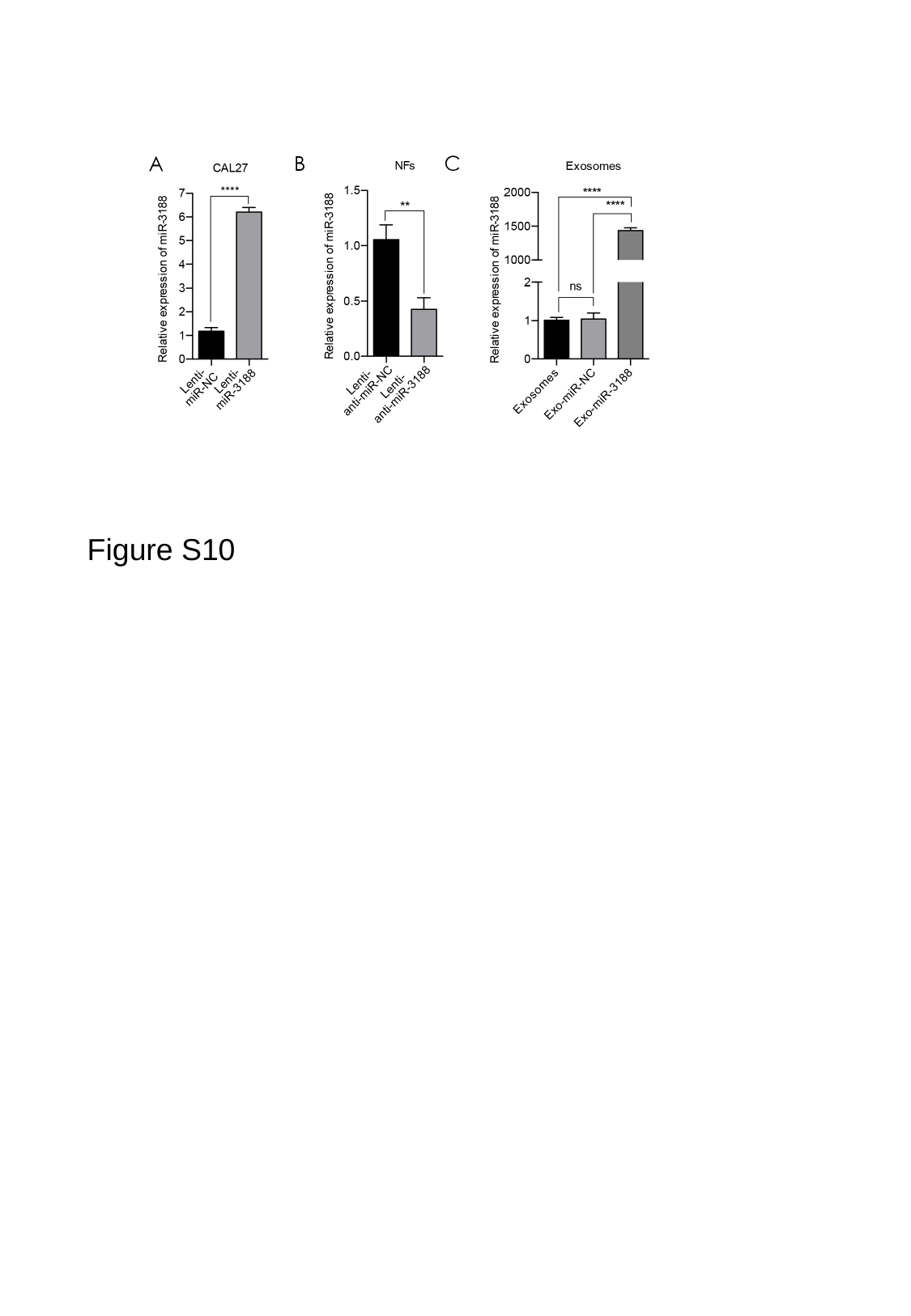

Figure S10

## Slide 11
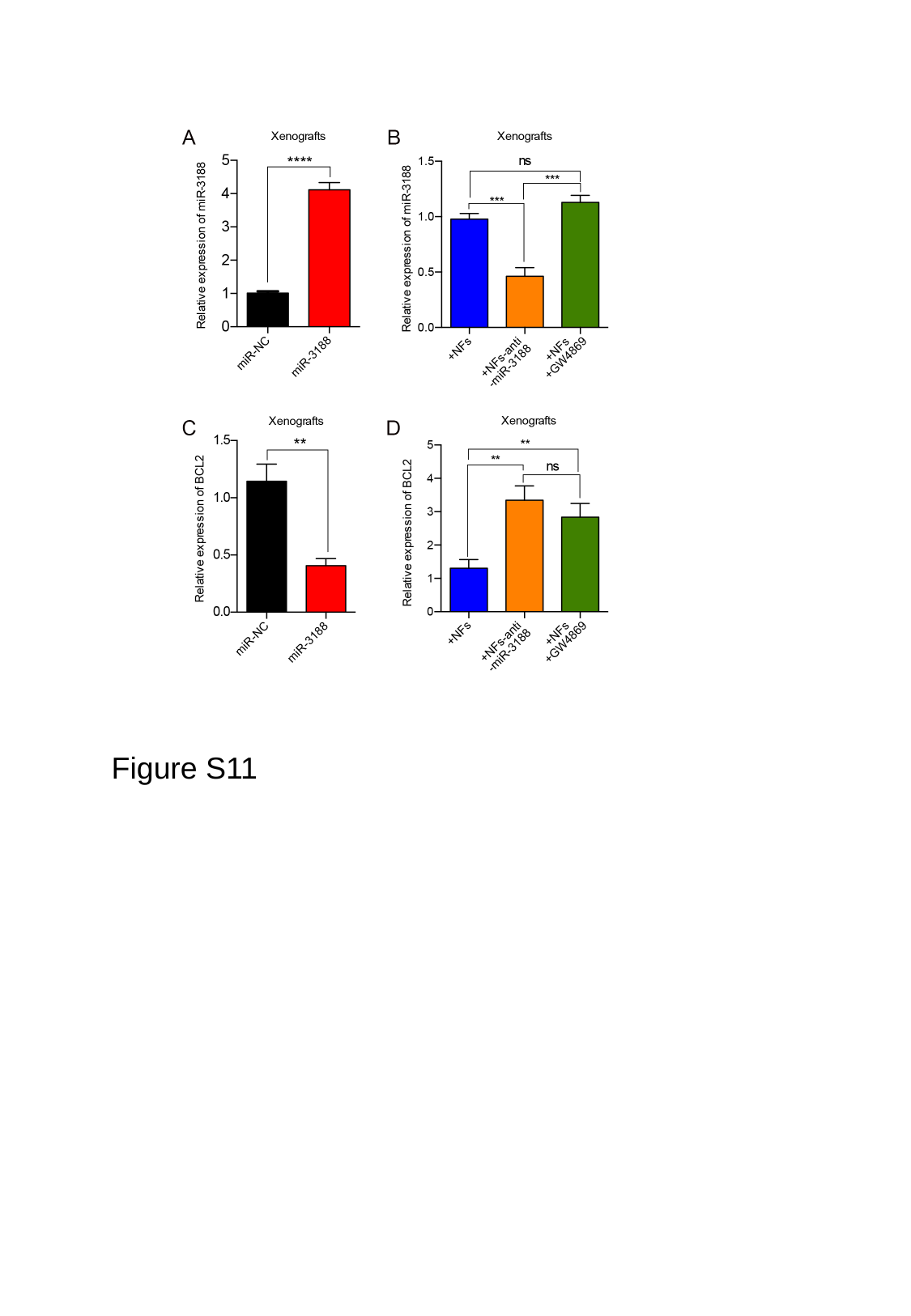

Figure S11

## Slide 12
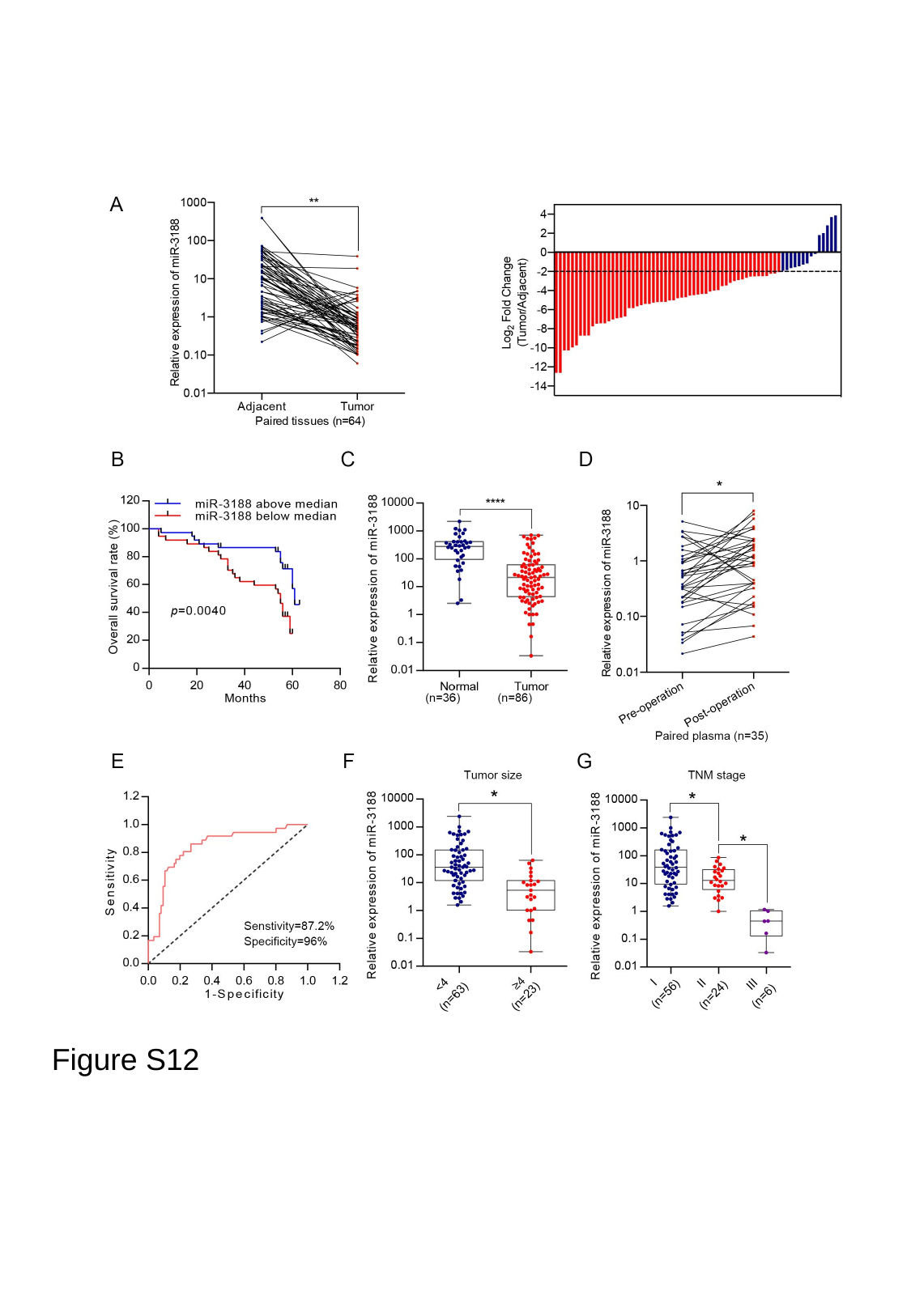

Figure S12

## Slide 13
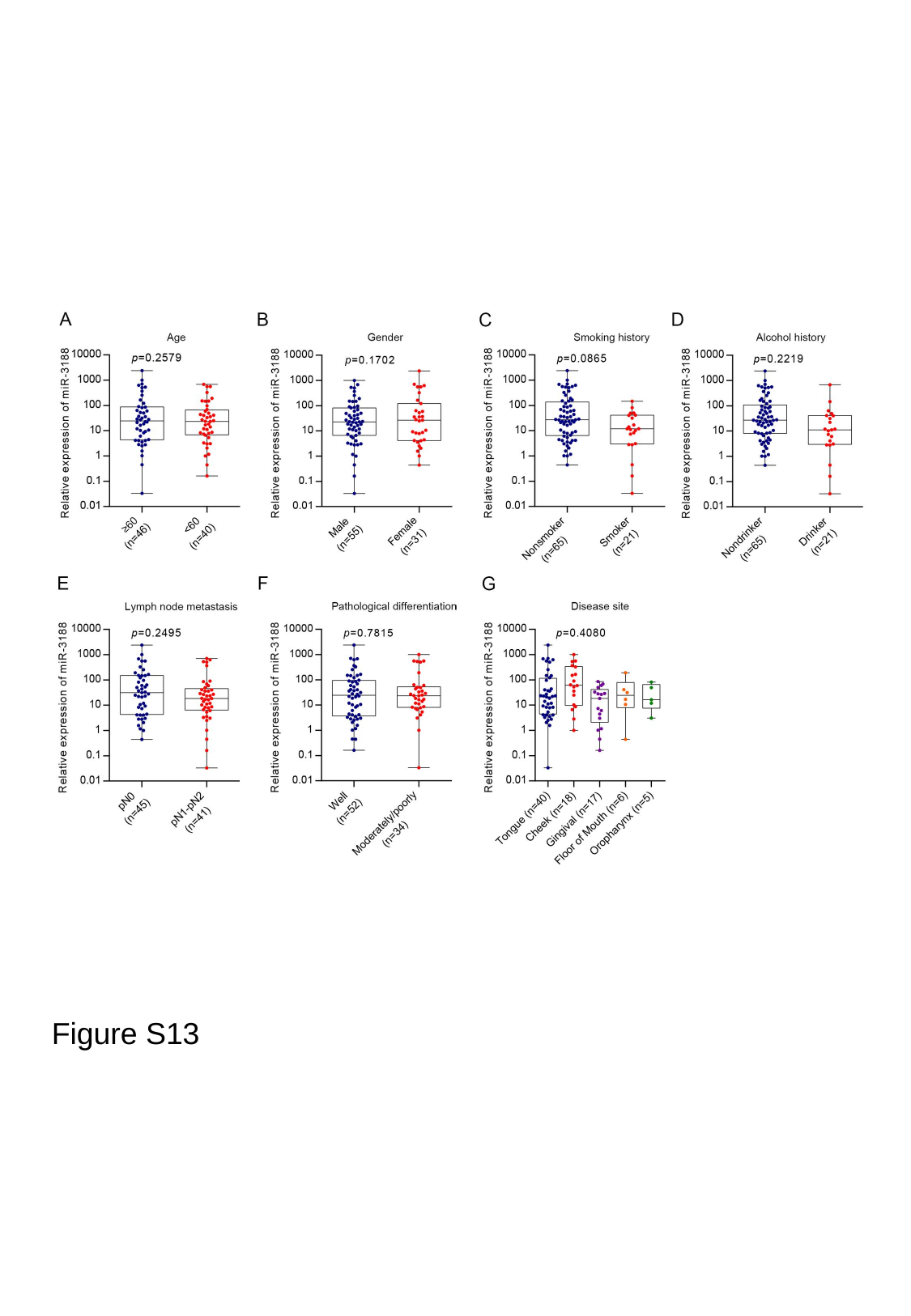

Figure S13
