# Supplementary material for: Loss of exosomal miR-3188 in cancer-associated fibroblasts contributes to HNC progression
Source: J Exp Clin Cancer Res. 2019 Apr 8;38:151. doi: 10.1186/s13046-019-1144-9 (PMC6454737; doi:10.1186/s13046-019-1144-9)
Supplement: Supplementary file 5 — Table S4. The differentially expressed miRNA in 3 pairs of NFs and CAFs. (DOC 234 kb) [file 13046_2019_1144_MOESM5_ESM.doc]

**Supplementary Table4. The differentially expressed miRNA in 3 pairs of** NFs and CAFs

| **miRNA IDs** | **p value** | **FC (CAF Vs NF)** | **Regulation** |
| --- | --- | --- | --- |
| hsa-let-7g-3p | 0.04233385 | 1.7157528 | up |
| hsa-miR-100-3p | 0.029072424 | 2.2308388 | up |
| hsa-miR-103a-2-5p | 0.00526467 | 2.1929355 | up |
| hsa-miR-10a-5p | 0.026457464 | 4.4012713 | up |
| hsa-miR-1285-3p | 0.048257135 | 1.6828854 | up |
| hsa-miR-140-5p | 0.016653687 | 2.4357936 | up |
| hsa-miR-196a-5p | 0.005965665 | 8.257619 | up |
| hsa-miR-3197 | 0.04435102 | 1.5266064 | up |
| hsa-miR-323a-3p | 0.28123748 | 2.1753697 | up |
| hsa-miR-335-5p | 0.047374077 | 5.8646417 | up |
| hsa-miR-338-5p | 0.030634822 | 1.6532743 | up |
| hsa-miR-3607-5p | 0.010880665 | 2.8844042 | up |
| hsa-miR-3620-3p | 0.0314742 | 2.1764817 | up |
| hsa-miR-365 | 0.034817807 | 2.1585972 | up |
| hsa-miR-369-5p | 0.023345582 | 3.00966 | up |
| hsa-miR-380-5p | 0.004959748 | 1.7184613 | up |
| hsa-miR-3918 | 0.038437515 | 2.0812302 | up |
| hsa-miR-3944-5p | 0.046692763 | 1.9644661 | up |
| hsa-miR-432-3p | 0.017630404 | 1.625322 | up |
| hsa-miR-4489 | 0.03128539 | 1.8873552 | up |
| hsa-miR-4496 | 0.049838655 | 1.6399802 | up |
| hsa-miR-4713-5p | 0.000381 | 1.6643194 | up |
| hsa-miR-485-3p | 0.0382405 | 1.5267447 | up |
| hsa-miR-660-5p | 0.04650969 | 1.8926601 | up |
| hsa-miR-7-5p | 0.048949666 | 2.7921956 | up |
| hsa-miR-922 | 0.006032263 | 1.7299054 | up |
| hsa-miR-1469 | 0.025758367 | -1.7978225 | down |
| hsa-miR-1915-3p | 0.00212397 | -1.7950555 | down |
| hsa-miR-2861 | 0.009582533 | -1.8806173 | down |
| hsa-miR-3187-3p | 0.008874454 | -2.248154 | down |
| hsa-miR-3188 | 0.04192311 | -2.3658204 | down |
| hsa-miR-335-3p | 0.028124895 | -2.1082973 | down |
| hsa-miR-3665 | 0.014094678 | -1.5088792 | down |
| hsa-miR-373-5p | 0.034928445 | -2.7231896 | down |
| hsa-miR-4434 | 0.013484819 | -2.070956 | down |
| hsa-miR-4466 | 0.04696144 | -1.8813907 | down |
| hsa-miR-4651 | 0.039852686 | -1.9057134 | down |
| hsa-miR-4674 | 0.03176096 | -1.9181929 | down |
| hsa-miR-4734 | 0.034645777 | -2.3844514 | down |
| hsa-miR-4741 | 0.01570065 | -2.2819982 | down |
| hsa-miR-4745-5p | 0.00529187 | -1.5814953 | down |
| hsa-miR-4758-5p | 0.023221768 | -1.8921623 | down |
| hsa-miR-4763-3p | 0.004965079 | -2.0607617 | down |
| hsa-miR-548 | 0.08205198 | -1.7532037 | down |
